# Enhancing pathogen detection in pediatric diarrheal stools: The case of molecular multiplexing with a real-time PCR assay

**DOI:** 10.1371/journal.pntd.0014823

**Published:** 2026-11-02

**Authors:** Goutam Chowdhury, Melissa Glenda Lewis, Alok Deb, Mamta Chawla-Sarkar, Sandipan Ganguly, Paulami Dutta, Santasabuj Das, Asish K. Mukhopadhyay, Thandavarayan Ramamurthy

**Affiliations:** ICMR-National Institute for Research in Bacterial Infections, Kolkata, India; University of Washington, UNITED STATES OF AMERICA

## Abstract

Diarrhea is a leading cause of pediatric morbidity and mortality worldwide, frequently caused by various bacterial, viral, and protozoan pathogens. Identification of these pathogens is important in clinical settings for proper management of the infection. Conventional stool culture technique is time-consuming for the detection of diarrheagenic pathogens. In addition, these traditional procedures lack sensitivity, require a specific laboratory structure, and well-trained staff. Conversely, multiplex molecular detection methods offer rapid results with less hands-on time and more sensitivity. This study compared the diagnostic performance of conventional microbiological methods and GastroFinder 2SMART multiplexed real-time PCR (real-time PCR) assay for detecting enteric pathogens in pediatric stool samples. Overall pathogen detection was significantly higher using this real-time PCR (81%) compared to the conventional assays (43.1%), reflecting differences in both sensitivity and pathogen panel breadth. Rotavirus, adenovirus, *Shigella*, and *Giardia lamblia* were frequently identified by conventional methods, whereas real-time PCR commonly detected adenovirus, *Campylobacter,* EPEC, ETEC, and norovirus. Certain pathogens such as *Yersinia enterocolitica*, *Clostridioides difficile*, STEC, and *Dientamoeba fragilis* were not tested in the conventional panel and therefore excluded for comparison. Sensitivity estimates varied, with lower sensitivity observed for EPEC, *Cryptosporidium, G. lamblia*, and rotavirus, partly due to the small sample numbers and wide confidence intervals. Specificity exceeded 90% for most pathogens, and diagnostic accuracy was > 90% for *Shigella*/EIEC, *Salmonella, Cryptosporidium*, *Giardia lamblia*, and astrovirus. Bayesian latent class analysis demonstrated the highest sensitivity for *Shigella*/EIEC (93.2%), followed by norovirus and adenovirus, while overall test performance, measured by the Youden Index, was greatest for *Shigella*/EIEC. These findings highlight the diagnostic use of real-time PCR and support its integration into routine diarrheal diagnostics to improve pathogen detection and clinical management.

## Author summary

Diarrhea remains a leading cause of childhood death in low-resource settings. Less sensitive conventional diagnostics methods often fail to identify the diarrheal pathogens. We compared a multiplex real-time PCR (GastroFinder 2SMART) assay with standard laboratory methods using stool specimens collected from 153 children with diarrhea. Using conventional diagnostic accuracy metrics and Bayesian latent class analysis—which estimates true test performance without assuming a perfect gold standard—we found that real-time PCR detected pathogens in 81% of samples versus 43.1% by conventional methods (p < 0.001). Detection improved substantially for bacteria (61% vs. 12%) and viruses (67% vs. 27%). Mixed infections were more common by the real-time PCR (75.8% vs. 15.7%). Performance varied by pathogen type: *Shigella*/EIEC showed excellent discriminatory ability (Youden Index 0.88; sensitivity 93.2%, specificity 95.4%), and negative predictive values exceeded 95% for most pathogens, indicating the use of GastroFinder 2SMART as a screening tool. However, rotavirus sensitivity was unexpectedly low (15%), a critical limitation for pediatric populations. These findings support the integration of molecular methods into routine diarrheal testing to improve pathogen detection and also highlight the need for additional rotavirus testing and careful pathogen-specific interpretation of results.

## Introduction

Globally, about 1.7 billion cases of childhood diarrheal infections occur each year, and it remains a leading cause of death in children below five years of age [1]. Diarrhea is caused by different bacterial, viral, and parasitic pathogens and their distribution and seasonality differ geographically. Considering its burden, early diagnosis and identification of etiological agents are important for appropriate diarrheal disease management, infection control, and prevention of outbreaks [2,3].

Most low- and middle-income countries (LMICs) do not use appropriate diagnostic tools in the health care system to identify the causative organism(s) to apply effective diarrheal treatment strategies. Moreover, the number of pathogens involved in diarrheal infections is many, and hence there is a need for the use of a broader test panel for their identification. In resolving this problem, the 76th World Health Assembly passed a resolution in May 2023 on strengthening diagnostic capacity [4]. In several multicentric studies, a combination of culture methods, PCR, ELISA, and microscopy has been used for the isolation and identification of different enteric pathogens [5,6]. Evidently, most of these assays are not highly sensitive; therefore, multiplex molecular tests are recommended for higher sensitivity and rapid turnaround time [7]. Molecular-based diagnostic systems are widely adopted in many high-income countries using several nucleic acid amplification techniques to reduce the time for the detection and improve the diagnostic quality [8–10].

This study aimed to: (i) evaluate the diagnostic performance of the GastroFinder 2SMART assay compared to conventional laboratory methods for detecting enteric pathogens in pediatric diarrheal stool samples; (ii) assess the sensitivity, specificity, predictive values, and clinical utility of the GastroFinder 2SMART assay; (iii) to test the Bayesian LCM diagnostic accuracy in the absence of a perfect gold standard, and (iv) to characterize the pathogen profile and mixed infection patterns in children with diarrhea in Kolkata, India.

## Materials and methods

### Ethics statement

This work was approved by the Institutional Ethical Committee (IEC) of the Indian Council of Medical Research-National Institute for Research in Bacterial Infections (ICMR-NIRBI), Kolkata, India (approval no. A-1/2015-IEC).

### Collection and processing of diarrheal stool specimens

The study was conducted during 2020–2022 at the Dr. B. C. Roy Memorial Hospital for Children, Kolkata, India, which is a major government pediatric hospital providing specialised care for infants and children. The hospital treats more than 9000 children suffering from diarrhea every year. Pediatric patients (children <5 years of age, consistent with WHO definitions for childhood diarrhea burden assessment) who had diarrhea were enrolled in this study. The rotavirus universal vaccination program was initiated in our region in 2019; however, due to the COVID-19 pandemic, rotavirus vaccination could not be effectively implemented during the study period. Age, gender, type of diarrhea, vomiting, fever, dehydration status, abdominal pain, and history of antibiotics were obtained from each enrolled patient. Using sterile catheters, stool samples were collected (approximately 5–10 g) in sterile containers. All samples were transported to the laboratory within 2 hrs and processed within 2–4 hrs of collection. Aliquot of stools was stored at -80°C for about 3 months before GastroFinder 2SMART testing. Stool samples were subjected to a maximum of two freeze-thaw cycles to maintain nucleic acid integrity.

This was a prospective diagnostic accuracy study based on consecutively enrolled eligible pediatric diarrheal stool specimens. All available eligible samples were included in the analysis. No formal a priori sample size calculation was performed because the study was designed as an initial evaluation of the diagnostic performance of the GastroFinder 2SMART test. Diagnostic accuracy estimates are therefore presented with 95% confidence intervals to indicate the precision of the estimates.

***Inclusion criteria:*** Pediatric patients aged <5 years presenting diarrhea (with or without fever), defined as the passage of three or more loose or watery stools within a 24-hour period, where diarrhea was the primary reason for seeking medical care, were enrolled in the study. Stool samples were collected after obtaining written informed consent from the parent or legal guardian and before the initiation of antibiotic therapy.

***Exclusion criteria:*** Children with a history of antibiotic use prior to stool sample collection, and children presenting with additional non-diarrheal diagnoses as the primary admission cause. Although children with a history of antibiotic use prior to stool sample collection were excluded, undisclosed antibiotic use cannot be ruled out. Stool samples that were inadequate in quantity, improperly collected, or not processed within the recommended time were also excluded.

During the study period, a total of 153 stool specimens were collected from pediatric patients presenting with acute diarrhea. As clinicians strictly evaluated and followed the sample collection criteria prior to sending the specimens to the laboratory, no samples required exclusion, and all 153 specimens were included in the final diagnostic evaluation. The conventional laboratory investigations represented the routine diagnostic workflow of our institute. Stool specimens were processed for culture by inoculation onto MacConkey, Hektoen enteric, and xylose lysine deoxycholate agar plates for isolation of bacteria representing Enterobacteriaceae; Butzler medium for *Campylobacter* spp. (BD Difco); and thiosulfate-citrate-bile salts-sucrose agar for *Vibrio* spp (Eiken). All the inoculated plates were incubated at 37°C for 18–24 hrs, except *Campylobacter* plates, which were incubated microaerobically for 48 hrs. Bacterial identification was made using biochemical tests including triple sugar iron agar, lysine iron agar, motility-indole-ornithine, citrate utilization, and urease tests, with serological confirmation for *Salmonella* and *Shigella* using specific antisera (Denka Seiken) [11]. For diarrheagenic *Escherichia coli*, multiplex PCR was performed targeting virulence genes: *elt* and *est* for enterotoxigenic *E. coli* (ETEC), *eae* for enteropathogenic *E. coli* (EPEC), and *aggR* for enteroaggregative *E. coli* (EAEC) [11]. For viral pathogens, total nucleic acid was extracted using the QIAamp Mini Kit (Qiagen) and reverse-transcription PCR was performed for rotavirus (VP6 gene), norovirus (RdRp and capsid regions), astrovirus, and sapovirus using published primer sets [12,13]. Adenovirus was detected by conventional PCR targeting the hexon gene. For parasite identification, stool samples were examined by direct wet mount microscopy and formalin-ethyl acetate concentration technique. Modified Ziehl-Neelsen staining was performed for *Cryptosporidium* and acid-fast oocysts. This conventional testing did not include *Yersinia enterocolitica*, *Clostridioides difficile*, STEC, or *Dientamoeba fragilis*, as these pathogens are infrequently encountered in our setting and were not part of the routine diagnostic algorithm.

### GastroFinder 2SMART real-time PCR assay

Only stool specimens were used for nucleic acid extraction with the QIAamp mini kit (Qiagen). Total nucleic acids were extracted according to the manufacturer's instructions and eluted in 50 μL of elution buffer, from which 5 μL was used in the GastroFinder 2SMART assay (PathoFinder). This multiplex one-step real-time PCR assay can detect 8 bacterial, 5 viral, and 4 parasitic pathogens in a single channel (S1 Table). One extraction blank and one no-template amplification control per batch were included to exclude laboratory contamination. *Shigella/*enteroinvasive *Escherichia coli* (EIEC) was detected using the *ipaH* target gene, which encodes an invasive plasmid antigen in both the pathogens.

The GastroFinder 2SMART assay combines normal PCR with melting curve analysis using ROX dye (6-carboxyl-X-Rhodamine) and Cy5 (Cyanine 5) dyes, and the results were inferred based on the multiple melting temperature (Tm) values recommended by the manufacturer. Normal PCR was performed using Veriti (Applied Biosystems) with the conditions of 50°C for 10 min, 95°C for 2 min, followed by 40 cycles of amplification consisting of 94°C for 20 sec, 55°C for 20 sec, 72°C for 35 sec. After this preamplification, real-time PCR was performed for generic amplification and detection in two steps using the LightCycler 480 (Roche Life Science) with initial denaturation at 95°C for 2 min followed by 10 cycles of 94°C for 15 sec, 55°C for 15 sec, 72°C for 15 sec. In the second step consists of 23 cycles with 94°C for 15 sec, 50°C for 15 sec, and 72°C for 15 sec with acquisition at 50°C. For melting curve analysis, denaturation 95°C for 2 min and incubation melting step 40°C for 90 sec. The results of the PCR assays were analyzed using the LightCycler software (Version 1.5.1). The assays were run with the internal controls and resutls were interpreted according to the manufacturers’ guidelines that correspond with the Tm value (S1 Table). Replicate testing was performed using randomly selected samples to confirm the result consistency.

For diagnostic performance evaluation, we classified samples as concordant (positive by both methods or negative by both methods) or discordant (positive by one method only). Sensitivity and specificity were calculated using conventional laboratory identification as the reference standard for each pathogen. In this context, ‘truly had a pathogen’ refers to detection by the reference conventional method. However, conventional methods may miss pathogens due to lower sensitivity, and hence this limitation is addressed through Bayesian latent class analysis, which is not a gold standard. The other diagnostic efficacy test parameters included in this study are sensitivity, specificity, positive predictive value (PPV), negative predictive value (NPV), and likelihood ratio (LR).

### Statistical methods

Data were entered into Excel (version 2021) and diagnostic accuracy calculations were performed using STATA 16 (StataCorp, 2019). To facilitate analysis of the diagnostic ability of GastroFinder 2SMART assay in detecting diarrheal pathogens, conventional laboratory identification was considered the reference standard. The clinical utility index (CUI) provides a measure of the practical usefulness of a diagnostic test in clinical practice [14]. CUI combines test accuracy with prevalence information and is calculated as: CUI+ = Sensitivity × PPV for positive tests, and CUI- = Specificity × NPV for negative tests. Grading thresholds for CUI follow established conventions: > 0.81 excellent, 0.64–0.80 good, 0.49–0.63 fair, 0.36–0.48 poor, and <0.36 very poor [14]. This index is particularly valuable for evaluating diagnostic tests in clinical settings as it indicates the proportion of clinically useful test results. The negative CUI (CUI-) correspondingly evaluates the utility of negative test results.

Diagnostic performance measures including sensitivity, specificity, predictive values, accuracy, and CUI were estimated with 95% confidence intervals (CI). Diagnostic accuracy was calculated as the proportion of correctly classified samples (true positives + true negatives/total samples), using conventional methods as the reference standard. The exact Clopper-Pearson confidence intervals were reported for sensitivity, specificity, and accuracy. Predictive values were calculated using the method described by Mercaldo et al. [15], except when values were equal to 0 or 100%, in which case the Clopper-Pearson confidence interval was applied.

For overall detection comparisons, each sample was classified as positive if at least one pathogen was detected by the respective method. To ensure comparability, only pathogens assessed by both methods were included in the analysis (McNemar’s test for paired proportions). In addition, category-specific detection rates (bacteria, viruses, and parasites) were calculated by grouping relevant pathogens. Because conventional laboratory methods may not represent a perfect gold standard, a Bayesian latent class model (LCM) was applied to estimate diagnostic accuracy without assuming a perfect reference test. True pathogen status was considered an unobserved latent variable. In this study, LCM assumes both the conventional method and the GastroFinder 2SMART assay are imperfect diagnostic tests, and sensitivity and specificity were estimated from the observed pattern of concordant and discordant test results. We assumed conditional independence between the two tests, indicating that, given the true infection status, the test results are independent. This is a commonly adopted assumption in Bayesian LCM for diagnostic test evaluation.

We fitted a two-test, one-population model assuming that the conditional independence between the two tests gives the true infection status. This assumption implies that conditional on true pathogen status, the result of one test does not depend on the result of the other. However, shared factors such as pathogen burden and specimen characteristics may introduce conditional dependence and were considered when interpreting the estimates. Non-informative β(0.5,0.5) prior distributions were specified for sensitivity, specificity, and disease prevalence. We ran two Markov chains for 50,000 iterations with a burn-in of 5,000 iterations and reported posterior median estimates with corresponding 95% credible intervals.

The Youden Index, calculated as sensitivity + specificity - 1, provides a single summary measure of overall test performance [16]. The index ranges from 0 (no discriminatory ability) to 1 (perfect discrimination), with higher values indicating better overall diagnostic performance. This index is useful for comparing test performance across different pathogens and for identifying the optimal threshold for positivity. Posterior medians with 95% credible intervals were reported, and the analysis was performed using JAGS implemented through R (rjags package).

## Results

A total of 153 diarrheal stool samples were collected from children below 5 years of age. The median age of the patients was 12 months (IQR 9,13) with almost an equal distribution of males (51.6%) and females (48.4%). In more than 96% of the patients, watery diarrhea was the most common clinical symptom, followed by vomiting (62.7%) and moderate dehydration (44.4%). Among the enrolled pediatric patients, metronidazole (45.1%), ciprofloxacin (23.5%), and ofloxacin (23.5%) were the most commonly used antimicrobials. The other clinical characteristics are presented in Table 1.

**Table 1:** Clinical characteristics of patients included in the study.

Clinical Features	Categories	n (%)
Age in months	Median (IQR)	12 (9, 13)
Gender	Male	79 (51.6)
	Female	74 (48.4)
Type of diarrhea	Watery	147 (96.1)
	Bloody & Mucoid	6 (3.9)
Vomiting	Yes	96 (62.7)
	No	57 (37.3)
Fever	Yes	9 (5.9)
	No	144 (94.1)
Dehydration	Severe	14 (9.2)
	Moderate	68 (44.4)
	None	71 (46.4)
Abdominal Pain	Yes	23 (15.1)
	No	130 (84.9)
Metronidazole	Yes	69 (45.1)
	No	84 (54.9)
Ornidazole	Yes	13 (8.5)
	No	140 (91.5)
Ciprofloxacin	Yes	36 (23.5)
	No	117 (76.5)
Ofloxacin	Yes	36 (23.5)
	No	117 (76.5)
Azithromycin	Yes	1 (0.7)
	No	152 (99.3)

As shown in Table 2, detection rates were significantly higher with real-time PCR than in conventional testing (81% vs 43.1%), particularly for bacterial (61% vs 15.7%) and viral pathogens (67.3% vs 27%). The majority of the discordant results were samples that were negative by conventional testing but positive by real-time PCR. In contrast, parasite detection rates were similar between the two methods. Sole infection caused by a single pathogen was observed in 58/66 (87.9%) of conventional test-positive samples, whereas real-time PCR detected a single pathogen in 30/124 (24.2%) of real-time PCR-positive samples. Mixed infections (two or more pathogens) were identified only in 8/66 (12.1%) of conventional test-positive samples but were substantially more frequent with real-time PCR, detected in 94/124 (75.8%) samples. When including pathogens not tested by conventional methods (*Y. enterocolitica*, *C. difficile*, STEC, and *D. fragilis*), the overall real-time PCR positivity was 131/153 (85.6%), with single infections in 37/131 (28.2%) and mixed infections in 94/131 (71.8%). Notably, GastroFinder 2SMART detected Shiga toxin-producing *E. coli* (STEC) in 25 samples (16.3%). STEC was not included in our routine conventional panel due to its rare representation in clinical samples from our region, and thus excluded from head-to-head performance comparisons. The difference in infection patterns between these two methods was statistically significant (p < 0.001).

**Table 2:** Comparison of conventional testing methods and real-time PCR results for the detection of pathogens.

Type of pathogen	Conventional	qPCR			p value
		Positive n (%)	Negative n (%)	Total n (%)	
Bacteria	Positive	15 (62.5)	9 (37.5)	**24 (15.7)**	<0.001
	Negative	78 (60.5)	51 (39.5)	129 (84.3)	
	**Total**	**93 (60.8)**	60 (39.2)	153	
Virus	Positive	30 (73.2)	11 (26.8)	**41 (26.8)**	<0.001
	Negative	73 (65.2)	39 (34.8)	112 (73.2)	
	**Total**	**103 (67.3)**	50 (32.7)	153	
Parasite	Positive	1 (11.1)	8 (88.9)	**9 (5.9)**	0.999
	Negative	8 (5.6)	136 (94.4)	144 (94.1)	
	**Total**	**9 (5.9)**	144 (94.1)	153	
Overall	Positive	55 (83.33)	11 (16.67)	**66 (43.1)**	<0.001
	Negative	69 (79.31)	18 (20.69)	87 (56.9)	
	**Total**	**124 (81.05)**	29 (19.0)	153	

Values are presented as n (%). Percentages in each row are calculated using the row total, whereas percentages in the total column are calculated using the overall study population (N = 153).

Table 3 shows the detection of enteric pathogens by conventional and real-time PCR methods. STEC (n = 25) and *D. fragilis* (n = 12) were also detected, but vibrios, *Y. enterocolitica* and *C. difficile* were not found. Except for vibrios, these pathogens were not included in the routine conventional diagnostic panel because they are infrequently encountered in our setting and were therefore excluded from the comparative performance analysis. In all real-time PCR-positive samples, an expected single melting peak has been observed with the corresponding pathogen and Tm values. Melting peaks were unambiguous; Tm distributions and replicate testing address potential borderline signals.

**Table 3:** Diagnostic performance of real-time PCR assays for detecting enteric pathogens compared with conventional laboratory identification.

Type of pathogen	Pathogen		Laboratory Identification		Sensitivity (95% CI)	Specificity (95% CI)	PPV (95% CI)	NPV (95% CI)	Accuracy (95% CI)	CUI + ^*^	CUI-^*^	p values^#^
			Positive	Negative								
**Bacteria**	*Shigella*/EIEC	Positive	10	10	100% (69.2-100)	93% (87.5-96.6)	50% (27.2-72.8)	100% (97.3-100)	93.46% (88.31-96.82)	0.500	0.930	0.002
		Negative	0	133								
	ETEC	Positive	1	36	100% (2.5-100)	76.3% (68.7-82.8)	2.7% (0.1-14.2)	100% (96.9-100)	76.47% (68.94-82.94)	0.027	0.763	<0.001
		Negative	0	116								
	*Campylobacter* spp	Positive	3	31	50% (11.8-88.2)	78.9% (71.4-85.2)	8.8% (1.9-23.7)	97.5% (92.8-99.5)	77.78% (70.36-84.09)	0.044	0.769	<0.001
		Negative	3	116								
	EPEC	Positive	0	36	0% (0-84.17)	76.2% (68.6-82.7)	0% (0-9.7)	98.3% (94-99.8)	75.16% (67.54-81.79)	0.000	0.749	<0.001
		Negative	2	115								
	*Salmonella* spp	Positive	1	12	100% (2.5-100)	92.1% (86.6-95.9)	7.7% (0.2-36)	100% (97.4-100)	92.16% (86.70-95.88)	0.077	0.921	<0.001
		Negative	0	140								
**Parasites**	*Cryptosporidium* spp	Positive	0	1	0% (0-97.5)	99.3% (96.4-100)	0% (0-97.5)	99.3% (96.4-100)	98.70% (95.39-99.84)	0.000	0.987	0.999
		Negative	1	152								
	*E. histolytica*	Positive	0	1	–	99.35% (96.4-99.9)	–	100% (97.6-100)	–	0.007	0.000	0.32
		Negative	0	151								
	*G. lamblia*	Positive	1	6	12.5% (0.3-52.7)	95.9% (91.2-98.5)	14.3% (0.4-57.9)	95.2% (90.4-98.1)	91.50% (85.91-95.40)	0.002	0.913	0.78
		Negative	7	139								
**Viruses**	Norovirus	Positive	7	22	87.5% (47.3-99.7)	84.8% (77.9-90.2)	24.1% (10.3-43.5)	99.2% (95.6-100)	84.97% (78.30-90.23)	0.211	0.841	<0.001
		Negative	1	123								
	Adenovirus	Positive	11	61	78.6% (49.2-95.3)	56.1% (47.5-64.5)	15.3% (7.9-25.7)	96.3% (89.6-99.2)	58.17% (49.93-66.08)	0.120	0.540	<0.001
		Negative	3	78								
	Rotavirus	Positive	3	0	15% (3.2-37.9)	100% (97.3-100)	100% (29.2-100)	88.7% (82.5-93.3)	88.9% (82.81-93.39)	0.150	0.887	<0.001
		Negative	17	133								
	Sapovirus	Positive	0	1	─	99.35% (96.4-99.9)	─	100% (97.6-100)	─	0.007	0.000	0.32
		Negative	0	152								
	Astrovirus	Positive	1	12	100% (2.5-100)	92.1% (86.6-95.9)	7.7% (0.2-36)	100% (97.4-100)	92.16% (86.70-95.88)	0.077	0.921	<0.001
		Negative	0	140								

Number in the parentheses indicate the 95% Confidence interval. CUI; Clinical utility index of the positive and negative test. > 0.81 excellent utility; 0.64–0.80 good utility 0.49–0.63 fair utility; 0.36–0.48; poor utility and <0.36 very poor utility; #McNemar’s test.

Sensitivity varied across pathogens. Sensitivity was 0% for EPEC (95%, CI: 0-84.2%) and *Cryptosporidium* spp. (95%, CI: 0-97.5%), 12.5% for *G. lamblia* (95%, CI: 0.3-52.7%), and 15% for rotavirus (95%, CI: 3.2-37.9), with wide confidence intervals due to a small number of positive samples (Table 3). In contrast, *Shigella*/EIEC demonstrated excellent sensitivity (100%, 95% CI: 69.2-100%) and high specificity (93%, 95% CI: 87.5-96.6%), resulting in greater diagnostic accuracy (93.5%). Norovirus also showed satisfactory performance with sensitivity of 87.5% (95%, CI: 47.3-99.7%) and specificity of 84.8% (95%, CI: 77.9-90.2%). The specificity exceeded 90% for several pathogens including *Shigella*/EIEC (93%), *Salmonella* spp. (92.1%), *Cryptosporidium* spp. (99.3%), *G. lamblia* (95.9%), and astrovirus (92.1%), with fairly precise confidence intervals (CI width 9%). The positive likelihood ratios (LR+) were >10 for *Shigella/*EIEC (14.3), *Salmonella* spp (12.7), and astrovirus (12.7), indicating strong diagnostic utility, whereas negative likelihood ratios (LR-) were ≤0.3 for *Shigella*/EIEC, ETEC, *Salmonella* spp., norovirus, and astrovirus confirming the assay's utility as a screening tool. As shown in Table 3, the CUI based on NPV was high (>0.80) for *Shigella*/EIEC, *Salmonella, Cryptosporidium*, *G. lamblia*, norovirus, and astrovirus, indicating excellent negative clinical utility. Overall, diagnostic accuracy exceeded 90% for *Shigella*/EIEC (93.5%), *Salmonella* (92.2%), *Cryptosporidium* (98.7%), *G. lamblia* (91.5%), and astrovirus (92.2%).

The results of Bayesian latent class analysis without a gold standard are presented in Table 4. High sensitivity of the PCR assay was seen in detecting *Shigella*/EIEC (93.2%), followed by norovirus (85.2%) and adenovirus (78.8%), with the lowest sensitivity in detecting rotavirus (16.7%). Specificity exceeded 90% for several pathogens including *Shigella*/EIEC*, Cryptosporidium*, *G. lamblia*, *Salmonella* spp, rotavirus, and astrovirus. The Youden Index was highest for *Shigella*/EIEC (0.88) and lowest for EPEC (0.02).

**Table 4:** Bayesian LCM estimates of diagnostic performance in evaluating the real-time PCR assay (95% Credible intervals).

Type of pathogen	Pathogen	Sensitivity % (95% CrI)	Specificity % (95% CrI)	Youden’s Index
**Bacteria**	*Shigella*/EIEC	93.2% (65.0-99.7)	95.4% (88.6-99.7)	0.88
	*Salmonella* spp.	60.0% (3.8-98.2)	93.3% (74.1-99.4)	0.53
	*Campylobacter* spp.	51.3% (8.0-95.7)	81.6% (45.1-97.9)	0.33
	ETEC	52.6% (4.2-97.9)	77.8% (26.2-97.0)	0.3
	EPEC	25.8% (1.3-90.9)	76.2% (20.8-96.4)	0.02
**Parasites**	*Cryptosporidium* spp.	22.1% (0.3-93.6)	99.0% (77.6-99.9)	0.21
	*G. lamblia*	17.7% (1.3-85.4)	96.2% (65.0-99.7)	0.14
**Viruses**	Norovirus	85.2% (51.4-99.2)	89.2% (80.4-99.2)	0.74
	Astrovirus	58.2% (2.9-98.1)	93.1% (52.8-99.3)	0.51
	Adenovirus	78.8% (47.5-98.0)	64.2% (49.3-96.6)	0.43
	Rotavirus	16.7% (4.0-53.3)	99.5% (97.0-100)	0.16

## Discussion

This study demonstrates that multiplex real-time PCR significantly enhances overall pathogen detection in pediatric diarrheal stools compared to conventional methods. However, the evaluation highlights a critical diagnostic weakness in the GastroFinder 2SMART assay: a severe lack of sensitivity for rotavirus, which limits its standalone utility in pediatric populations where rotavirus is a leading pathogen. The overall detection rate increased from 43.1% with conventional testing to 81% with real-time PCR, showing nearly a 2-fold increase. This substantial rise was primarily driven by enhanced detection of bacterial and viral pathogens, while parasite detection rates remained comparable between methods. Mixed infections were identified in 75.8% of real-time PCR-positive samples compared with 12.1% of conventional test-positive samples, suggesting that polymicrobial infections are substantially more common in the patient population. This observation was consistent in other investigations [17,18].

The high negative predictive value (>95%) observed for most pathogens indicates that the assay could serve as an effective screening tool to rule out infectious etiologies. However, translating these diagnostic gains into improved antimicrobial stewardship depends on several operational factors, including rapid test turnaround times, sampling prior to empiric administration, clinician willingness to de-escalate therapy based on negative molecular results, and institutional antibiotic guidelines [10]. However, the clinical efficacy of this assay requires further investigation, its application has the potential to improve the diagnosis of infectious gastroenteritis. Molecular detection of STEC highlights an under-recognized pathogen burden in pediatric diarrhea in Eastern India. Because molecular panels detect toxin genes without differentiating active infection from low-level colonization, quantitative load assessment and local epidemiological validation remain necessary. The low rotavirus detection rate by the real-time PCR assay represents a critical limitation in this study. Rotavirus remains one of the leading causes of severe dehydrating diarrhea in children under 5 years globally, accounting for 42.2% of deaths in this age group [3]. In India, rotavirus is estimated to cause approximately 78,000 deaths annually among children under five years of age, and has been a priority target for the National Immunization Program since 2016 [19]. The substantially lower sensitivity of this assay for rotavirus compared to conventional RT-PCR (which targets VP6) suggests that the NSP3 gene target used in the real-time PCR assay may be suboptimal for clinical detection. NSP3 is a non-structural protein expressed at lower levels during infection compared to the structural VP6 protein, which may result in lower detection sensitivity, particularly when multiple targets are multiplexed [20]. Furthermore, the higher Tm values observed (62–65) indicate low target abundance or potential competition for reagents in the multiplex reaction.

Clinical implications of this underdiagnosis are significant. False-negative rotavirus results could lead to: (1) inappropriate antibiotic use to control diarrhea, contributing to antimicrobial resistance; (2) failure to implement appropriate infection control measures, increasing transmission risk in healthcare settings; (3) missed opportunities for rotavirus vaccine efficacy monitoring; and (4) underestimation of the true rotavirus disease burden in community and hospital settings. For these reasons, clinicians should consider supplementing this assay with a VP6-targeting test (either ELISA or RT-PCR) when clinical suspicion of rotavirus is high, or seek alternative multiplex assays that include robust VP6-targeting primers.

The distribution of enteric pathogens in our study population showed both similarities and notable differences compared to other geographical regions. Norovirus and adenovirus (20.3%) were the most frequently detected viral pathogens, which is consistent with findings from recent studies in India [12] and other South Asian countries [21]. However, the rotavirus detection rate (2.0%) was substantially lower than the 25–40% typically reported in the pre-vaccination period [22], reflecting the assay limitations. Among bacterial pathogens, *Campylobacter* spp. and *Shigella*/EIEC were predominant, similar to findings from MAL-ED cohort sites in Bangladesh and Pakistan [2]. In contrast, studies from African sites (Côte d'Ivoire and Mali) have reported a higher prevalence of EAEC and *Campylobacter* [23], while *Salmonella* was more commonly detected in Southeast Asian settings [24]. These geographical variations highlight the importance of region-specific diagnostic panels and the need for local epidemiological data to inform clinical practice. Notably, GastroFinder 2SMART detected STEC in 25 samples (16.3%). While STEC was not included in our routine conventional panel and thus excluded from head-to-head performance comparisons. Its frequent molecular detection highlights an under-recognized pathogen burden in pediatric diarrhea in Eastern India. However, because molecular panels detect toxin genes without differentiating active infection from low-level colonization, quantitative load assessment and local epidemiological validation remain necessary.

Since the conventional diagnostic algorithm reflected routine clinical practice in our region, direct comparisons could only be performed for pathogens routinely investigated by existing laboratory methods. In this study, the diagnostic performance of the tested real-time PCR assay varied substantially across pathogen types. For bacterial pathogens, the assay demonstrated excellent sensitivity for *Shigella*/EIEC (100% vs conventional, 93.2% by LCM) and moderate sensitivity for *Campylobacter* (50% vs conventional, 51.3% by LCM) and ETEC (100% vs conventional, 52.6% by LCM). These findings align with previous evaluations of multiplex gastrointestinal panels, which have reported sensitivities ranging from 89-100% for bacterial pathogens [25,26]. The high specificity (>90%) and positive likelihood ratios (>10) for *Shigella/*EIEC indicate that a positive result effectively confirms infection, with strong diagnostic utility for clinical decision-making. The excellent NPV (>97%) for all bacterial pathogens suggests that negative results effectively rule out these infections, supporting the assay's use as a screening tool. The detection of pathogens not routinely tested in conventional panels (*Y. enterocolitica*, *C. difficile*, STEC, and *D. fragilis*) adds value to the assay, as these organisms are often overlooked despite their clinical significance.

For viral pathogens, norovirus showed moderate sensitivity (sensitivity 87.5%, specificity 84.8%) relative to single-target RT-PCR, indicating that while yield is improved, a subset of norovirus infections may still escape multiplex detection due to competitive inhibition between different primers that sometimes reduce overall diagnostic sensitivity compared to simplex RT-qPCR assays [27]. The moderate performance for adenovirus (sensitivity 78.6%, specificity 56.1%) is similar to previous reports of reduced specificity in multiplex panels, wherein symptomatic pediatric cohorts primarily reflect prolonged viral shedding from past infections or low target copy numbers detected by PCR but below the detection threshold of conventional assays [9]. The poor sensitivity for rotavirus (15%) and sapovirus (0%) highlights the need for alternative or supplementary tests for these pathogens [28]. For parasitic pathogens, specificity was excellent (>95%) for all parasites, indicating that positive results are reliable. However, sensitivity was poor for *Cryptosporidium* spp. (0%) and *G. lamblia* (12.5%), likely reflecting the high parasite load required for detection in multiplex reactions and competition for reagents. These findings suggest that dedicated parasitological methods like microscopy or single-target PCR remain necessary for parasite diagnosis in clinical settings.

The discordance between conventional and molecular methods warrants careful consideration. Only 10% of intestinal bacteria can be cultured using selective media [29]. For bacterial pathogens, low culture detection rates might reflect several factors: selective media may not support the growth of all strains. Molecular methods on the other hand detect both viable and non-viable organisms, and the detection rate is twofold higher than culture methods [30]. The moderate concordance observed for some viral pathogens is concerning and may be attributed to target mismatch between assays. Conventional viral methods used simplex/multiplex PCR targeting different gene regions than the real-time PCR test, which could explain some discordance. Additionally, fecal specimens contain residual inhibitors such as heme, bilirubin, bile salts, and complex carbohydrates that reduce PCR efficiency by interacting with target DNA/RNA or inactivating Taq polymerases [25,31]. These findings underscore that conventional methods are inadequate reference standards. While Bayesian models rely on conditional independence assumptions, other robust validation strategies can identify the limitations and ensure reliable estimates [32].

The Bayesian latent class analysis should be interpreted in the context of its underlying assumptions. Although we assumed conditional independence between the conventional methods and multiplex real-time PCR, given the true infection status, this assumption may not be adequate in routine clinical practice. Both these methods are influenced by pathogen burden, while culture-based methods may additionally be affected by bacterial viability and recent antimicrobial exposure. The Bayesian estimates remain dependent on model assumptions and should be interpreted cautiously, particularly for low prevalence pathogens.

We observed substantial concordance between the frequentist estimates (using conventional methods as the reference standard) and Bayesian latent class analysis estimates for most pathogens. For *Shigella*/EIEC, both approaches yielded excellent sensitivity and specificity, although Bayesian estimates were associated with wider uncertainty. Similarly, Norovirus estimates were almost consistent, indicating robust detection across analytic methods. The Youden Index from Bayesian analysis ranked *Shigella*/EIEC highest, corroborating conventional accuracy metrics.

However, notable discrepancies were observed for pathogens with low prevalence in the study population. For *Salmonella* spp., astrovirus, and *Campylobacter*, Bayesian estimates of sensitivity were lower than frequentist estimates, with wide credible intervals reflecting uncertainty due to few positive samples. These findings indicate the value of the latent class approach in situations where the reference standard is imperfect, as it provides complementary estimates by accounting for the limitations of both testing methods.

The discordance between approaches was most pronounced for pathogens where conventional methods are known to have low sensitivity. In these cases, the frequentist approach may underestimate true sensitivity, while the Bayesian approach provides a more conservative but likely more accurate estimate. For clinical decision-making, we endorse using the Bayesian provides alternative estimates for pathogens where conventional methods are unreliable, but caution that the wide credible intervals for low-prevalence pathogens limit their interpretative value. Because the conventional diagnostic algorithm reflected routine clinical practice in our region, direct comparisons could only be performed for pathogens routinely investigated by conventional laboratory methods.

The application of multiplex molecular diagnostics in resource-limited settings requires careful consideration of cost, infrastructure, and clinical utility. While conventional microbiology requires manual effort and specialized expertise, its basic equipment infrastructure is widely available and low-cost. Conversely, implementing multiplex molecular assays like GastroFinder 2SMART requires substantial upfront capital for multi-channel real-time PCR thermocyclers, uninterrupted power supply, continuous cold-chain infrastructure, and expensive reagent kits. Thus, in many LMIC settings, molecular panels are currently more feasible as centralized referral diagnostics rather than point-of-care tools. The estimated cost per test may be substantially higher than conventional methods but less comparable to other commercial multiplex panels.

In field scenarios, the high NPV of the assay (>95% for most pathogens) could support a “rule-out” strategy, where negative results guide clinicians to consider non-infectious causes of diarrhea and avoid unnecessary antibiotics. However, the low sensitivity for rotavirus and parasites means that negative results do not reliably exclude these pathogens, potentially leading to underdiagnosis. The high rate of mixed infections detected raises important questions about clinical management: it remains unclear whether all detected pathogens are contributing to disease, and whether treatment should target all or only the most clinically significant pathogens. In the absence of quantitative thresholds, clinicians may face diagnostic uncertainty.

The low prevalence of fever among the hospitalized patients reflects that the primary presentation is acute watery diarrhea without systemic signs of infection. Enrolled patients presented with moderate or severe diarrhea as their main symptom, and the inclusion criteria specifically required diarrhea as the primary cause for seeking medical care. The low fever rate is consistent with the high proportion of watery diarrhea rather than inflammatory or invasive diarrhea, which is more commonly associated with fever. Patients were not enrolled in this study if diarrhea was a secondary complaint or incidental finding. This clinical context is important for LMIC settings where syndromic management is common, as it underscores the need for accurate diagnostics to differentiate viral from bacterial etiologies and avoid unnecessary antibiotic use.

### Limitations of this study

This study has several important limitations that should be considered when interpreting the findings. First, the sample size was comparatively small, particularly for evaluating rare pathogens, resulting in wide confidence intervals for several estimates. Second, conventional methods were used as the reference standard for diagnostic accuracy calculations, but these methods are inadequate and may underestimate true pathogen prevalence. While we addressed this using Bayesian latent class analysis, which accounts for weak reference standards, the conditional independence assumption may not fully hold for all pathogens. Third, we did not perform orthogonal confirmation for all real-time PCR-positive samples. Fourth, the tested real-time PCR does not relate pathogen-load thresholds for defining positivity; therefore, some mixed infections may represent low-level detections of colonizing organisms rather than true active infections, potentially overestimating the polymicrobial infection. Fifth, the study was conducted in a single geographic region, which may limit generalizability to other settings with different pathogen distributions and epidemiological characteristics. Sixth, we did not systematically evaluate the cost-effectiveness of the assay compared to conventional methods, which is a critical consideration for implementation in resource-limited settings. Seventh, vaccination status was not available for all participants due to the COVID-19 pandemic, limiting our ability to assess the impact of rotavirus vaccination on detection rates. Eighth, the clinical relevance of detecting multiple pathogens remains uncertain, as it is unclear whether all detected organisms are contributing to disease. Finally, because the conventional diagnostic algorithm reflected routine clinical practice in our region, direct comparisons could only be performed for pathogens routinely investigated by conventional laboratory methods, which may limit the comprehensiveness of our performance evaluations for less commonly tested organisms.

## Conclusion

The GastroFinder 2SMART multiplex real-time PCR assay demonstrates substantial analytical capacity for enhancing pathogen detection in pediatric diarrheal stools, with an overall detection rate 2-fold higher than conventional methods. The assay shows high diagnostic sensitivity for selected bacterial targets like *Shigella*/EIEC, supporting its use in clinical settings. High negative predictive values observed for most pathogens indicate their utility as a screening tool to rule out infectious etiologies and support antimicrobial stewardship. However, the assay has critical limitations, most notably the marked underdiagnosis of rotavirus, which precludes its use as a stand-alone diagnostic for pediatric diarrhea. When implemented alongside supplementary rotavirus testing and careful pathogen-specific interpretation, molecular multiplexing can substantially improve diarrheal disease surveillance and clinical management in hospital settings.

## Supporting information

S1 Table Target pathogens and Tm values of the GastroFinder 2SMART assay. (DOCX)
