# Supplementary material for: Enhancing pathogen detection in pediatric diarrheal stools: The case of molecular multiplexing with a real-time PCR assay
Source: PLoS Negl Trop Dis. 2026 Nov 2;20(11):e0014823. doi: 10.1371/journal.pntd.0014823 (PMC13750648; doi:10.1371/journal.pntd.0014823)
Supplement: Supplementary file 1 [file pntd.0014823.s001.docx]

**S1 Table .** Target pathogens and Tm values of the GastroFinder 2SMART assay

| **qPCR assay** | **Probe label** | **Pathogen (target gene)** | **Average *Tm* value** | ***Tm* range** |
| --- | --- | --- | --- | --- |
| 1 | ROX | *Campylobacter* spp (16S-rRNA) | 55.0 | 53.5-56.5 |
|  |  | *Clostridioides difficile* (toxin B) | 61.0 | 59.5-62.5 |
|  |  | *Salmonella* spp (*invA*) | 65.5 | 63.5-67.0 |
|  |  | *Yersinia enterocolitica* (*gyrB*) | 69.0 | 67.5-70.5 |
|  | Cy5 | Enteropathogenic *E. coli*, EPEC (*eae*) | 53.0 | 51.5-54.5 |
|  |  | Enteroinvasive *E. coli/Shigella* spp (*ipaH*) | 56.5 | 55.0-58.0 |
|  |  | Enterotoxigenic *E. coli* (*elt/est*) | 63.5 | 62.0-65.0 |
|  |  | Shiga toxin-producing *E. coli* (*sxt1/sxt2*) | 68.5 | 66.5-70.5 |
| 2 | ROX | *Entamoeba histolytica* (18S-rRNA) | 55.0 | 53.5-56.5 |
|  |  | *Giardia lamblia (*18S-rRNA) | 61.0 | 59.5-62.5 |
|  |  | *Cryptosporidium* spp (oocyst wall protein) | 65.5 | 63.5-67.0 |
|  |  | *Dientamoeba fragilis* (18S-rRNA) | 69.0 | 67.5-70.5 |
|  |  | *Clostridioides difficile* (toxin A) | 78.5 | 77.0-80.0 |
|  | Cy5 | Adenoviurs (Hexon H) | 53.0 | 51..5-54.5 |
|  |  | Astrovirus (Capsid protein ORF-2) | 56.5 | 55.0-58.0 |
|  |  | Rotavirus (NSP3) | 63.5 | 62.0-65.0 |
|  |  | Norovirus (Capsid protein ORF-1) | 67.5 | 65.5-69.5 |
|  |  | Sapovirus (Polymerase Capsid protein ORF-1) | 73.0 | 71.5-74.5 |
