# Supplementary material for: Enhancing pathogen detection in pediatric diarrheal stools: The case of molecular multiplexing with a real-time PCR assay
Source: PLoS Negl Trop Dis. 2026 Nov 2;20(11):e0014823. doi: 10.1371/journal.pntd.0014823 (PMC13750648; doi:10.1371/journal.pntd.0014823)
Supplement: Supplementary file 2 [file pntd.0014823.s003.docx]

**Response to the Reviewer’s comments**

Manuscript title: Enhancing Pathogen Detection in Pediatric Diarrheal Stools: The Case of Molecular Multiplexing with a Real-time PCR Assay

PLOS Neglected Tropical Diseases

Manuscript PNTD-D-26-00570:

**Editorial comments**

If the reviewer comments include a recommendation to cite specific previously published works, please review and evaluate these publications to determine whether they are relevant and should be cited. There is no requirement to cite these works unless the editor has indicated otherwise.

**Response:** Reviewers not suggested to cite any additional works

1. Please ensure that the CRediT author contributions listed for every co-author are completed accurately and in full.
   At this stage, the following Authors/Authors require contributions: Goutam Chowdhury, Melissa Glenda Lewis, Alok Deb, Mamta Chawla-Sarkar, Sandipan Ganguly, Paulami Dutta, Santasabuj Das, Asish K. Mukhopadhyay, and Thandavarayan Ramamurthy. Please ensure that the full contributions of each author are acknowledged in the "Add/Edit/Remove Authors" section of our submission form.
   The list of CRediT author contributions may be found here: <https://journals.plos.org/plosntds/s/authorship#loc-author-contributions>

**Response:** We have included the CRediT author contributions for all authors (Page 17). The contributions are listed as follows:

**Conceptualization:** Thandavarayan Ramamurthy, Asish K. Mukhopadhyay

**Data curation:** Goutam Chowdhury, Melissa Glenda Lewis, Alok Deb, Mamta Chawla-Sarkar, Thandavarayan Ramamurthy

**Formal analysis:** Goutam Chowdhury, Melissa Glenda Lewis, Mamta Chawla-Sarkar, Sandipan Ganguly, Paulami Dutta, Thandavarayan Ramamurthy, Asish K. Mukhopadhyay

**Funding acquisition:** Santasabuj Das,

**Project administration:** Alok Deb, Santasabuj Das, Asish K. Mukhopadhyay

**Writing – original draft** Thandavarayan Ramamurthy, Asish K. Mukhopadhyay:

**Writing – review & editing:** Goutam Chowdhury, Melissa Glenda Lewis, Alok Deb, Mamta Chawla-Sarkar, Santasabuj Das,

2. Please provide an Author Summary. This should appear in your manuscript between the Abstract (if applicable) and the Introduction, and should be 150-200 words long. The aim should be to make your findings accessible to a wide audience that includes both scientists and non-scientists. Sample summaries can be found on our website under Submission Guidelines:
   <https://journals.plos.org/plosntds/s/submission-guidelines#loc-parts-of-a-submission>

**Response:** We have added the following Author Summary between the Abstract and Introduction sections (Page 3).

**Author Summary:**

Diarrhea remains a leading cause of childhood death in low-resource settings. Less sensitive conventional diagnostics methods often fail to identify the diarrheal pathogens. We compared a multiplex real-time PCR (GastroFinder 2SMART) assay with standard laboratory methods in 153 children with diarrhea. Using conventional diagnostic accuracy metrics and Bayesian latent class analysis—which estimates true test performance without assuming a perfect gold standard—we found that GastroFinder 2SMART detected pathogens in 85.6% of samples versus 43.1% by conventional methods (p<0.001). Detection improved substantially for bacteria (61% vs. 12%) and viruses (67% vs. 27%). Mixed infections were more common by GastroFinder 2SMART (87.9% vs. 12.1%). Performance varied by pathogen: Shigella/EIEC showed excellent discriminatory ability (Youden Index 0.88; sensitivity 93.2%, specificity 95.4%), and negative predictive values exceeded 95% for most pathogens, indicating the use of GastroFinder 2SMART as a screening tool. However, Rotavirus sensitivity was unexpectedly low (15-16.7%), a critical limitation for pediatric populations. These findings support integrating molecular methods into routine diarrheal testing to improve pathogen detection and also highlight the need for additional rotavirus testing and careful pathogen-specific interpretation of results.

3. We do not publish any copyright or trademark symbols that usually accompany proprietary names, eg ©, ®, or TM (e.g. next to drug or reagent names). Therefore please remove all instances of trademark/copyright symbols throughout the text, including: - ® on pages: 2, 3, 4, 5, 6, 9, 10, 11, 18, 19, and 25.

**Response:** We have deleted all trademark/copyright symbol throughout the manuscript.

4. Please upload all main figures as separate Figure files in .tif or .eps format. For more information about how to convert and format your figure files please see our guidelines:
   <https://journals.plos.org/plosntds/s/figures>

**Response:** As suggested by the Editor and the reviewers, we have removed the Figures

5. We notice that your supplementary Tables are included in the manuscript file. Please remove them and upload them with the file type 'Supporting Information'. Please ensure that each Supporting Information file has a legend listed in the manuscript after the references list.

**Response:** In the revised manuscript, we have removed the supplementary table and uploaded as ‘Supporting Information’. Legend for this supporting information has been included after the reference list

6. In the online submission form, you indicated that We are ready to share any additional data asked by the reviewers.. All PLOS journals now require all data underlying the findings described in their manuscript to be freely available to other researchers, either
   1. In a public repository
   2. Within the manuscript itself
   3. Uploaded as supplementary information.
   This policy applies to all data except where public deposition would breach compliance with the protocol approved by your research ethics board. If your data cannot be made publicly available for ethical or legal reasons (e.g., public availability would compromise patient privacy), please explain your reasons by return email and your exemption request will be escalated to the editor for approval. Your exemption request will be handled independently and will not hold up the peer review process, but will need to be resolved should your manuscript be accepted for publication. One of the Editorial team will then be in touch if there are any issues.

**Response:** Additional data/information suggested by the editors and reviewers are provided within the revised manuscript.

7. Please amend your detailed Financial Disclosure statement. This is published with the article. It must therefore be completed in full sentences and contain the exact wording you wish to be published.
   i) State the initials, alongside each funding source, of each author to receive each grant. For example: "This work was supported by the National Institutes of Health (####### to AM; ###### to CJ) and the National Science Foundation (###### to AM)."
   ii) State what role the funders took in the study. If the funders had no role in your study, please state: "The funders had no role in study design, data collection and analysis, decision to publish, or preparation of the manuscript."
   iii) If any authors received a salary from any of your funders, please state which authors and which funders..
   If you did not receive any funding for this study, please simply state: u201cThe authors received no specific funding for this work.u201d

**Response:** We have amended Financial Disclosure statement with all the details. All the authors received the fund from the Indian Council of Medical Research and hence we have stated statement as “This work was supported by the Indian Council of Medical Research under the grant ID: ER/FBS/PHASE-II/2023/DR/MD. The funders had no role in study design, data collection and analysis, decision to publish, or preparation of the manuscript”. (Page 17)

8. We have amended your Competing Interest statement to comply with journal style. We kindly ask that you double check the statement and let us know if anything is incorrect.

**Response:** We have amended the Competing Interest statement as, “The authors have declared that no competing interests exist.” (Page 17)

**REVIEWER’S COMMENTS**

**Methods**

**Reviewer # 1**

The objectives are articulated (though 'accuracy' is not listed initially, and pops up later). The design and population are appropriate. The sample size seems reasonable. Ethics approval was obtained.

**Response**: We have revised the Introduction section to clearly state the study objectives at the beginning (Lines 109 to 114).

“This study aimed to: (i) evaluate the diagnostic performance of the GastroFinder 2SMART assay compared to conventional laboratory methods for detecting enteric pathogens in pediatric diarrheal stool samples; (ii) assess the sensitivity, specificity, predictive values, and clinical utility of the GastroFinder 2SMART assay; (iii) to test the Bayesian LCM diagnostic accuracy in the absence of a perfect gold standard, and (iv) to characterize the pathogen profile and mixed infection patterns in children with diarrhea in Kolkata, India”.

1. Line 86 – could there please be MUCH more information provided on what the standard stool workup was (what agar plates, what incubation, what identification, specifics of the diarrhoeagenic E coli molecular testing, specifics of the viral PCR workup, and more details of the microscopy techniques)?

**Response:** In the revised manuscript, we have included conventional laboratory methods with detailed information on culture media, incubation conditions, identification procedures, and PCR testing protocol for the detection of diarrheagenic *Escherichia coli* (Lines 145 to 165). The outline of these methods is given below.

The conventional laboratory investigations represented the routine diagnostic workflow of our institute. Stools specimens were processed for culture by inoculation onto MacConkey, Hektoen enteric, and xylose lysine deoxycholate agar plates for isolation of bacteria representing Enterobacteriaceae; Butzler medium for *Campylobacter* spp. (BD Difco); and thiosulfate-citrate-bile salts-sucrose agar for *Vibrio* spp (Eiken). All the inoculated plates were incubated at 37°C for 18-24 hrs, except *Campylobacter* plates, which were incubated microaerobically for 48 hrs. Bacterial identification was made using biochemical tests including triple sugar iron agar, lysine iron agar, motility-indole-ornithine, citrate utilization, and urease tests, with serological confirmation for *Salmonella* and *Shigella* using specific antisera (Denka Seiken) [10]. For diarrheagenic *Escherichia coli*, multiplex PCR was performed targeting virulence genes: *elt* and *est* for enterotoxigenic *E. coli* (ETEC), *eae* for enteropathogenic *E. coli* (EPEC), and *aggR* for enteroaggregative *E. coli* (EAEC) [10]. For viral pathogens, total nucleic acid was extracted using the QIAamp Mini Kit (Qiagen) and reverse-transcription PCR was performed for rotavirus (VP6 gene), norovirus (RdRp and capsid regions), astrovirus, and sapovirus using published primer sets [11, 12]. Adenovirus was detected by conventional PCR targeting the hexon gene. For parasite identification, stool samples were examined by direct wet mount microscopy and formalin-ethyl acetate concentration technique. Modified Ziehl-Neelsen staining was performed for *Cryptosporidium* and acid-fast oocysts. This conventional testing did not include *Yersinia enterocolitica*, *Clostridioides difficile*, STEC, or *Dientamoeba fragilis*, as these pathogens are infrequently encountered in our setting and were not part of the routine diagnostic algorithm.

2. What was the interval between sample collection and storage at -80C? How long were samples stored before testing?

**Response:** Stool samples were processed within 2-4 hrs of collection. After processing, aliquots of stools were stored at -80°C for about 3 months before GastroFinder 2SMART testing. Stool samples were subjected to a maximum of two freeze-thaw cycles to maintain nucleic acid integrity. We have added this information to the Methods section (Lines 124 to 128).

3. Line 118 – I do not understand what this means. If a true positive is defined as both assays positive, then both conventional and 2SMART testing would both have 100% sensitivity for all targets, since both would have detected all true positives. This is contradicted by Line 135. Line 138 says that PPV was the probability that patients with a positive test 'truly had a pathogen' – what does 'truly had a pathogen' mean?

**Response:** Sorry for this confusion. We have clarified the definition in the revised manuscript. The "two-by-two rule" is used to classify samples for calculating diagnostic performance metrics, not to define true positives for sensitivity calculation. With the details given below, we have revised this section for clarity (Lines from 193 to 201).

" For diagnostic performance evaluation, we classified samples as concordant (positive by both methods or negative by both methods) or discordant (positive by one method only). Sensitivity and specificity were calculated using conventional laboratory identification as the reference standard for each pathogen. In this context, 'truly had a pathogen' refers to detection by the reference conventional method. However, conventional methods may miss pathogens due to lower sensitivity, and hence this limitation is addressed through Bayesian latent class analysis, which is not a gold standard. The other diagnostic efficacy test parameters included in this study are sensitivity, specificity, positive predictive value (PPV), negative predictive value (NPV), and likelihood ratio (LR)”.

4. I have real concerns about the validity of the conventional reference standard assays. It only detected 1/37 ETEC positives, 0/36 EPEC positives, 1/12 Salmonella positives, 3/34 Campylobacter positives, 7/29 Norovirus positives, and 11/72 adenovirus positives, for example. For the bacterial pathogens, this is lower than I would have expected (even given the expected superior sensitivity of molecular testing) and – for the viral pathogens – why was there such a difference if molecular is currently being used for conventional testing?

**Response:** We acknowledge that conventional methods have inherent limitations, which is precisely why molecular testing is being evaluated. We have expanded the discussion to address these discrepancies (Lines 392 to 405).

The discordance between conventional and molecular methods warrants careful consideration. Only 10% of intestinal bacteria can be cultured using selective media [24]. For bacterial pathogens, low culture detection rates might reflect several factors: selective media may not support the growth of all strains. Molecular methods on the other hand detect both viable and non-viable organisms, and the detection rate is twofold higher than culture methods [25]. The moderate concordance observed for some viral pathogens is concerning and may be attributed to target mismatch between assays. Conventional viral methods used simplex/multiplex PCR targeting different gene regions than the real-time PCR test, which could explain some discordance. Additionally, fecal specimens contain residual inhibitors such as heme, bilirubin, bile salts, and complex carbohydrates that reduce PCR efficiency by interacting with target DNA/RNA or inactivating Taq polymerases [26]. These findings underscore that conventional methods are inadequate reference standards. While Bayesian models rely on conditional independence assumptions, other robust validation strategies can identify the limitations and ensure reliable estimates [27].

5. What is the CUI, and how is it helpful to gauge performance of the assay of interest.

**Response:** We have added a clear explanation of the Clinical Utility Index (CUI) in the Methods section (Lines 206 to 216).

The clinical utility index (CUI) provides a measure of the practical usefulness of a diagnostic test in clinical practice. CUI combines test accuracy with prevalence information and is calculated as: CUI+ = Sensitivity × PPV for positive tests, and CUI- = Specificity × NPV for negative tests. This index is particularly valuable for evaluating diagnostic tests in clinical settings as it indicates the proportion of clinically useful test results. The negative CUI (CUI-) correspondingly evaluates the utility of negative test results. This metric helps clinicians understand whether test results are likely to influence patient management decisions. A positive CUI (CUI+) > 0.81 indicates excellent clinical utility, indicating the test provides actionable positive results for most patients; 0.64-0.80 specifies good utility; 0.49-0.63 fair utility; 0.36-0.48 poor utility; and <0.36 very poor utility.

6. How does the Bayesian latent class analysis (and the Youden index) add to our understanding of the assay of interest? This probably needs to be explained in details for readers like me who are not familiar with this analysis.

**Response:** In the Methods section of the revised manuscript, we have provided a clear description of the Bayesian latent class analysis (LCM) and Youden index. The newly added information is as follows (Lines 235 to 258).

Because conventional laboratory methods may not represent a perfect gold standard, a Bayesian latent class model (LCM) was applied to estimate diagnostic accuracy without assuming a perfect reference test. True pathogen status was considered an unobserved latent variable. In this study, LCM assumes both the conventional method and the GastroFinder 2SMART assay are imperfect diagnostic tests, and sensitivity and specificity were estimated from the observed pattern of concordant and discordant test results. We assumed conditional independence between the two tests, indicating that, given the true infection status, the test results are independent. This is a commonly adopted assumption in Bayesian LCM for diagnostic test evaluation. We fitted a two-test, one-population model assuming that the conditional independence between the two tests gives the true infection status. This assumption implies that conditional on true pathogen status, the result of one test does not depend on the result of the other. However, shared factors such as pathogen burden and specimen characteristics may introduce conditional dependence and were considered when interpreting the estimates. Non-informative β(0.5,0.5) prior distributions were specified for sensitivity, specificity, and disease prevalence. We ran two Markov chains for 50,000 iterations with a burn-in of 5,000 iterations and reported posterior median estimates with corresponding 95% credible intervals.

The Youden Index, calculated as Sensitivity + Specificity - 1, provides a single summary measure of overall test performance. The index ranges from 0 (no discriminatory ability) to 1 (perfect discrimination), with higher values indicating better overall diagnostic performance. This index is useful for comparing test performance across different pathogens and for identifying the optimal threshold for positivity. Posterior medians with 95% credible intervals were reported, and the analysis was performed using JAGS implemented through R (rjags package).

7. Could you please describe the setting in more detail? Number of yearly clinic visits, number of yearly hospitalizations

**Response:** We have added the following detailed information about the study setting (Lines 117 to 128).

The study was conducted during 2020-2022 at the Dr. B. C. Roy Memorial Hospital for Children, Kolkata, India, which is a major government pediatric hospital providing specialised care for infants and children. The hospital treats more than 9000 children suffering from diarrhea every year. Pediatric patients (children <5 years of age, consistent with WHO definitions for childhood diarrhea burden assessment) who had diarrhea were enrolled in this study. Age, gender, type of diarrhea, vomiting, fever, dehydration status, abdominal pain, and history of antibiotics were obtained from each enrolled patient. Using sterile catheters, stool samples were collected (approximately 5-10 g) in sterile containers. All samples were transported to the laboratory within 2 hrs and processed within 2-4 hrs of collection. Aliquot of stools was stored at -80°C for about 3 months before GastroFinder 2SMART testing. Stool samples were subjected to a maximum of two freeze-thaw cycles to maintain nucleic acid integrity.

8. What does 'pediatric' mean? Age 1 day to 18 years?

**Response:** We have clarified the age definition in the Materials and Methods section (Lines 120 to 122).

Pediatric patients (children <5 years of age, consistent with WHO definitions for childhood diarrhea burden assessment) who had diarrhea were enrolled in this study. A

9. How was it decided whether to take a rectal swab or bulk stool?

**Response:** In the Methods section, we have briefly added information about the collection of stool specimens (Lines 123 to 124). Rectal swabs samples were not used in this study.

Using sterile catheters, stool samples were collected (approximately 5-10 g) in sterile containers.

10. What type of rectal swabs were used? What transport medium?

**Response:** We have collected only the bulk stool specimens in this study.

11. How was diagnostic accuracy calculated? Is this really helpful given the expected low sensitivity of conventional testing?

**Response:** Diagnostic accuracy was calculated as the proportion of correctly classified samples (true positives + true negatives/total samples), using conventional methods as the reference standard. This sentence was included in the Methods section (under Statistical Methods; lines 218 to 220).

We recognize that this approach has limitations given the known lower sensitivity of conventional methods. However, these methods remain the routine diagnostic approach in most clinical laboratories, particularly in resource-limited settings. Making comparison against these methods provides clinically relevant information regarding how new molecular tests would perform in current diagnostic practice.

To address the limitations of imperfect reference standards, we also performed Bayesian latent class analysis, which estimates diagnostic performance without assuming that conventional methods represent a perfect gold standard.

**Reviewer #2: -**Are the objectives of the study clearly articulated with a clear testable hypothesis stated?

**Comments:** The objective was clear. A testable hypothesis was not provided - yet the authors do compare the conventional assay to the multiplex qPCR

**Response:** As shown below, the objectives of this study have been included in the revised manuscript.

This study aimed to: (i) evaluate the diagnostic performance of the GastroFinder 2SMART assay compared to conventional laboratory methods for detecting enteric pathogens in pediatric diarrheal stool samples; (ii) assess the sensitivity, specificity, predictive values, and clinical utility of the GastroFinder 2SMART assay; (iii) to test the Bayesian LCM diagnostic accuracy in the absence of a perfect gold standard, and (iv) to characterize the pathogen profile and mixed infection patterns in children with diarrhea in Kolkata, India. (Lines 109 to 114).

We would like to submit here that these objectives overall cover the testable hypotheses also.

**Comments**: Is the study design appropriate to address the stated objectives?
The study design is not described including the study setting. One only learns the design as they read the different sections of the manuscript and from the objective.

**Response:** We have added the following detailed information about the study setting (Lines 117 to 123).

“The study was conducted during 2020-2022 at the Dr. B. C. Roy Memorial Hospital for Children, Kolkata, India, which is a major government pediatric hospital providing specialised care for infants and children. The hospital treats more than 9000 children suffering from diarrhea every year. Pediatric patients (children <5 years of age, consistent with WHO definitions for childhood diarrhea burden assessment) who had diarrhea were enrolled in this study. Age, gender, type of diarrhea, vomiting, fever, dehydration status, abdominal pain, and history of antibiotics were obtained from each enrolled patient.”

**Comments**: Is the population clearly described and appropriate for the hypothesis being tested?
Details of the study participants are quite scanty for any meaningful reconstruction of the of the study. The study location(s) is not provided including which hospital(s) the participants were drawn from and the type of facility. Participants usage of antibiotics or other treatments prior to sample collection is not provided.

**Response:** We have included suggested information regarding study setting, patient’s information and the type of clinical information obtained in lines 117 to 123.

**Comments:** -Is the sample size sufficient to ensure adequate power to address the hypothesis being tested?
Sample size details are not provided - to get a clear understanding of how many would have been required to test for the pathogens. On line 46-48 - the authors point “Sensitivity estimates varied, with lower sensitivity observed for EPEC, Cryptosporidium, G. lamblia, and Rotavirus, partly due to the small sample numbers and wide confidence intervals.” Additionally, for the 153 individual patients- how many contributed stool and how many contributed swabs?

**Response:** We thank the reviewer for asking for the details of sample size calculations required to evaluate a test kit. First, we would like to mention that all the clinical specimens tested in this study are bulk stools and not the swabs. We acknowledge that the sample size limited the precision of estimates for pathogens with low prevalence, resulting in wider confidence intervals, and have clarified this as a limitation in the revised manuscript. We have also included the inclusion and exclusion criteria of patients in this study (Lines 129 to 144)

This was a prospective diagnostic accuracy study based on consecutively enrolled eligible pediatric diarrheal stool specimens. All available eligible samples (n = 153) were included in the analysis. No formal a priori sample size calculation was performed because the study was designed as an initial evaluation of the diagnostic performance of the GastroFinder 2SMART test. Diagnostic accuracy estimates are therefore presented with 95% confidence intervals to indicate the precision of the estimates.

**Inclusion criteria:** Pediatric patients aged <5 years presenting diarrhea (with or without fever), defined as the passage of three or more loose or watery stools within a 24-hour period, were enrolled in the study. Stool samples were collected after obtaining written informed consent from the parent or legal guardian and before the initiation of antibiotic therapy.

**Exclusion criteria:** Children aged >5 years, those with a history of antibiotic use prior to stool sample collection, those without diarrhea, and those whose parents or legal guardians declined to provide informed consent were excluded. Although children with a history of antibiotic use prior to stool sample collection were excluded, undisclosed antibiotic use cannot be ruled out. Stool samples that were inadequate in quantity, improperly collected or not processed within the recommended time were also excluded.

We have also modified the Limitation section as shown below (Lines 462 to 485)

This study has several important limitations that should be considered when interpreting the findings. First, the sample size was comparatively small, particularly for evaluating rare pathogens, resulting in wide confidence intervals for several estimates. Second, conventional methods were used as the reference standard for diagnostic accuracy calculations, but these methods are inadequate and may underestimate true pathogen prevalence. While we addressed this using Bayesian latent class analysis, which accounts for weak reference standards, the conditional independence assumption may not fully hold for all pathogens. Third, we did not perform orthogonal confirmation for all real-time PCR-positive samples. Fourth, the tested real-time PCR does not relate pathogen-load thresholds for defining positivity; therefore, some mixed infections may represent low-level detections of colonizing organisms rather than true active infections, potentially overestimating the polymicrobial infection. Fifth, the study was conducted in a single geographic region, which may limit generalizability to other settings with different pathogen distributions and epidemiological characteristics. Sixth, we did not systematically evaluate the cost-effectiveness of the assay compared to conventional methods, which is a critical consideration for implementation in resource-limited settings. Seventh, vaccination status was not available for all participants due to the COVID-19 pandemic, limiting our ability to assess the impact of rotavirus vaccination on detection rates. Eighth, the clinical relevance of detecting multiple pathogens remains uncertain, as it is unclear whether all detected organisms are contributing to disease. Finally, because the conventional diagnostic algorithm reflected routine clinical practice in our region, direct comparisons could only be performed for pathogens routinely investigated by conventional laboratory methods, which may limit the comprehensiveness of our performance evaluations for less commonly tested organisms.

**Comments**: Were correct statistical analysis used to support conclusions?
I have limitations in statistics - but assume that Bayesian latent class model (LCM) as applied to estimate diagnostic accuracy in the study to generates Posterior medians with 95% credible intervals. On line 150-152 authors indicate “This method considers both the tests as imperfect. We adopted a two-test one-population model assuming conditional independence. For all the parameters, we assumed a non-informative prior distribution (beta distribution (0.5,0.5))”. The authors need to be clear about potential independence in detection of nucleic acids by both approaches/assays, dependence on pathogen load, effect of recent antibiotic use on culture positivity etc which may bias estimates. The limitation of applications of such LCM approaches when applied to pathogen detection work needs careful considerations related to inherent aspects of samples sizes, pathogen prevalences, model identification, priors and assumptions.

**Response:** We thank the reviewer for this thoughtful and constructive comment. We agree that Bayesian latent class models rely on several assumptions when estimating diagnostic accuracy in the absence of a perfect reference standard. In the revised manuscript we have clarified the assumptions. We have also expanded the discussion to acknowledge that the estimates may be influenced by factors such as pathogen burden, organism viability, recent antimicrobial exposure, relatively small sample size, and the low prevalence of several pathogens. We further emphasize that the Bayesian latent class analysis was performed as a complementary analysis to address the absence of a perfect reference standard and should be interpreted alongside the conventional diagnostic accuracy measures rather than as a replacement for them.

In view of all the above descriptions, we have included the following in respective sections.

**Methods:** Because conventional laboratory methods may not represent a perfect gold standard, a Bayesian latent class model (LCM) was applied to estimate diagnostic accuracy without assuming a perfect reference test. True pathogen status was considered an unobserved latent variable. In this study, LCM assumes both the conventional method and the GastroFinder 2SMART assay are imperfect diagnostic tests, and sensitivity and specificity were estimated from the observed pattern of concordant and discordant test results. We assumed conditional independence between the two tests, indicating that, given the true infection status, the test results are independent. This is a commonly adopted assumption in Bayesian LCM for diagnostic test evaluation. We fitted a two-test, one-population model assuming that the conditional independence between the two tests gives the true infection status. This assumption implies that conditional on true pathogen status, the result of one test does not depend on the result of the other. However, shared factors such as pathogen burden and specimen characteristics may introduce conditional dependence and were considered when interpreting the estimates. Non-informative β(0.5,0.5) prior distributions were specified for sensitivity, specificity, and disease prevalence. We ran two Markov chains for 50,000 iterations with a burn-in of 5,000 iterations and reported posterior median estimates with corresponding 95% credible intervals. (Lines 235 to 251).

**Discussion:** The Bayesian latent class analysis should be interpreted in the context of its underlying assumptions. Although we assumed conditional independence between the conventional methods and multiplex real-time PCR, given the true infection status, this assumption may not be adequate in routine clinical practice. Both these methods are influenced by pathogen burden, while culture-based methods may additionally be affected by bacterial viability and recent antimicrobial exposure. The Bayesian estimates remain dependent on model assumptions and should be interpreted cautiously, particularly for low prevalence pathogens. (Lines 407 to 413).

**Limitations:** This study has several important limitations that should be considered when interpreting the findings. First, the sample size was comparatively small, particularly for evaluating rare pathogens, resulting in wide confidence intervals for several estimates. Second, conventional methods were used as the reference standard for diagnostic accuracy calculations, but these methods are inadequate and may underestimate true pathogen prevalence. While we addressed this using Bayesian latent class analysis, which accounts for weak reference standards, the conditional independence assumption may not fully hold for all pathogens. Third, we did not perform orthogonal confirmation for all real-time PCR-positive samples. Fourth, the tested real-time PCR does not relate pathogen-load thresholds for defining positivity; therefore, some mixed infections may represent low-level detections of colonizing organisms rather than true active infections, potentially overestimating the polymicrobial infection. Fifth, the study was conducted in a single geographic region, which may limit generalizability to other settings with different pathogen distributions and epidemiological characteristics. Sixth, we did not systematically evaluate the cost-effectiveness of the assay compared to conventional methods, which is a critical consideration for implementation in resource-limited settings. Seventh, vaccination status was not available for all participants due to the COVID-19 pandemic, limiting our ability to assess the impact of rotavirus vaccination on detection rates. Eighth, the clinical relevance of detecting multiple pathogens remains uncertain, as it is unclear whether all detected organisms are contributing to disease. Finally, because the conventional diagnostic algorithm reflected routine clinical practice in our region, direct comparisons could only be performed for pathogens routinely investigated by conventional laboratory methods, which may limit the comprehensiveness of our performance evaluations for less commonly tested organisms. (Lines 463 to 485).

**Other concerns**

1. Does the amount of starting material differ for nucleic acid extractions considering both swabs and whole stool were used? Would this influence the down-stream assays to be conducted? From a practical perspective - a swab from a diarrhoea stool sample with unusual character - line 84-85 (liquid, watery, mucous or bloody purulent) may influence how much material is collected. Since this was a “head-to-head” comparison (i.e. GastroFinder® 2SMART kit compared with conventional assay techniques - lines 76-79) was one swab split between the different targets and assays? Could this influence study outcomes because of bias considering the metrics for success - performance, sensitivity, specificity, positive predictive value (PPV), negative predictive value (NPV), and likelihood ratio (LR)?

**Response:** We thank the reviewer for this important comment. We apologize for the lack of clarity in the manuscript. Although the original text referred to "stool samples/rectal swabs," only stool specimens were included in the present evaluation and used for both the conventional laboratory methods and the GastroFinder 2SMART test. Rectal swabs were not used in this study. We have revised the Materials and Methods section to explicitly state that only stool samples were analyzed and that aliquots from the same stool specimen were used for the respective conventional and molecular assays.

2. Two references are provided for the routine tests for enteric bacteria by culture, PCR assay for diarrheagenic Escherichia coli, reverse-transcription PCR for enteric viruses, and microscopy for the identification of parasites (line 86-88). The study by Nair et al 2010 https://pmc.ncbi.nlm.nih.gov/articles/PMC2901208/, indicates “Faecal specimens were collected in McCartney bottles using sterile catheters or as rectal swabs in Cary Blair medium and were examined within 2 hrs for 24 enteric pathogens comprising bacterial, viral and parasitic pathogens using a combination of conventional, immunological and molecular methods” and don’t highlight use of fecal swabs. The other by Mitra et al., 2020 https://pubmed.ncbi.nlm.nih.gov/32585545/ (targeted 4 common enteric viruses (rotavirus, norovirus, adenovirus, astrovirus) but due to paywall - I could not access the methods. Conventional methods for certain pathogens such as Salmonella spp and Dientamoeba falgillis are not detailed in the study by Mitra et al are not provided. It’s also not clear why there was no effort to target the specific pathogens present in the GastroFinder® 2SMART assay - on line 44 - 46 - authors indicate “Certain pathogens, including Yersinia enterocolitica, Clostridioides difficile, STEC, and Dientamoeba fragilis were not tested in the conventional panel and therefore not considered discordant”. There is no clear explanation in the methods as to why this is the case yet the authors, concurrently, provide quite some details of the PCR from line 103 to 117 on the GastroFinder® 2SMART assay is combined with a normal PCR.

**Response:** We agree that the conventional diagnostic methods and the rationale for the pathogen panel require further clarification. In the revised manuscript, we have expanded the Materials and Methods section to briefly describe the conventional laboratory methods used for bacterial culture, PCR for diarrheagenic *Escherichia coli*, RT-PCR for enteric viruses, and microscopy for parasites, while retaining the original references describing these validated protocols.

We have also clarified that this study was designed as a real-world comparison between the GastroFinder® 2SMART assay and the routine diagnostic workflow available at our Institution during the study period. Consequently, several pathogens included in the multiplex qPCR panel, such as *Yersinia enterocolitica*, *Clostridioides difficile*, STEC, and *Dientamoeba fragilis*, were not routinely investigated by conventional methods because they are infrequently encountered in our region and were not part of the standard diagnostic algorithm. Therefore, these pathogens were excluded from the head-to-head comparative analysis and were not considered discordant results. This clarification has now been added to the Materials and Methods, Results and Discussion sections.

**Methods:** The conventional laboratory investigations represented the routine diagnostic workflow of our institute. Stool specimens were processed for culture by inoculation onto MacConkey, Hektoen enteric, and xylose lysine deoxycholate agar plates for isolation of bacteria representing Enterobacteriaceae; Butzler medium for *Campylobacter* spp. (BD Difco); and thiosulfate-citrate-bile salts-sucrose agar for *Vibrio* spp (Eiken). All the inoculated plates were incubated at 37°C for 18-24 hrs, except *Campylobacter* plates, which were incubated microaerobically for 48 hrs. Bacterial identification was made using biochemical tests including triple sugar iron agar, lysine iron agar, motility-indole-ornithine, citrate utilization, and urease tests, with serological confirmation for *Salmonella* and *Shigella* using specific antisera (Denka Seiken) [10]. For diarrheagenic *Escherichia coli*, multiplex PCR was performed targeting virulence genes: *elt* and *est* for enterotoxigenic *E. coli* (ETEC), *eae* for enteropathogenic *E. coli* (EPEC), and *aggR* for enteroaggregative *E. coli* (EAEC) [10]. For viral pathogens, total nucleic acid was extracted using the QIAamp Mini Kit (Qiagen) and reverse-transcription PCR was performed for rotavirus (VP6 gene), norovirus (RdRp and capsid regions), astrovirus, and sapovirus using published primer sets [11, 12]. Adenovirus was detected by conventional PCR targeting the hexon gene. For parasite identification, stool samples were examined by direct wet mount microscopy and formalin-ethyl acetate concentration technique. Modified Ziehl-Neelsen staining was performed for *Cryptosporidium* and acid-fast oocysts. This conventional testing did not include *Yersinia enterocolitica*, *Clostridioides difficile*, STEC, or *Dientamoeba fragilis*, as these pathogens are infrequently encountered in our setting and were not part of the routine diagnostic algorithm. (Lines 145 to 165)

**New Reference:**

Oka T, Yamamoto SP, Iritani N, Sato S, Tatsumi C, Mita T, Yahiro S, Shibata S, Wu FT, Takagi H. Polymerase chain reaction primer sets for the detection of genetically diverse human sapoviruses. Arch Virol. 2020;165(10):2335-2340. doi: 10.1007/s00705-020-04746-9.

**Results:** **Table 3** shows the detection of enteric pathogens by conventional and real-time PCR methods. STEC (n=25) and *D. fragilis* (n=12), were also detected, but vibrios, *Y. enterocolitica* and *C. difficile* were not found. Except for vibrios, these pathogens were not included in the routine conventional diagnostic panel because they are infrequently encountered in our setting and were therefore excluded from the comparative performance analysis. (Lines 283 to 287)

**Discussion:** Since the conventional diagnostic algorithm reflected routine clinical practice in our region, direct comparisons could only be performed for pathogens routinely investigated by existing laboratory methods. In this study, the diagnostic performance of the tested real-time PCR assay varied substantially across pathogen types. (Lines 366 to 369)

3. Does the nucleic acids extraction kits matter? The QIAamp DNA Mini Kit (Qiagen) was used for extraction of total nucleic acids and eluted in a volume of 50 μl of which 5 μl was used for PCR. There are minimal details on the procedure for extractions including whether bead beating was a necessary step considering the nature and biology of bacterial pathogens etc. The manufacturer indicates that the “validated extraction methods NucliSENS® easyMAG® (bioMérieux)” - which may simply imply that the manufacturer has not tested the Qiagen kit - so, providing extra details of the kits protocol is likely essential - and whether this would vary by swabs compared to whole stool. Sometimes, knowing the amount of microbial load maybe important - for example 16S for bacterial pathogens. Would the Kit be efficient at targeting RNA based viruses?

**Response:** We have revised the Materials and Methods section to clarify that only stool specimens, and not rectal swabs, were used in this study.

**Methods:** Only stool specimens were used for nucleic acid extraction with the QIAamp mini kit (Qiagen). Total nucleic acids were extracted according to the manufacturer's instructions and eluted in 50 μL of elution buffer, from which 5 μL was used in the GastroFinder 2SMART assay (PathoFinder). (Lines 170 to 173)

Total nucleic acids were extracted from stool using the QIAamp Mini Kit (Qiagen) according to the manufacturer's protocol and subsequently used for the GastroFinder 2SMART test. The extraction procedure yielded nucleic acids suitable for the detection of both DNA- and RNA-based pathogens, as demonstrated by the successful detection of RNA viruses, including rotavirus, norovirus, and astrovirus. The objective of this study was to evaluate the diagnostic performance of the GastroFinder 2SMART test under our routine laboratory workflow rather than to compare different nucleic acid extraction methods. We therefore did not evaluate the performance of alternative extraction platforms or quantify microbial load. These points have now been clarified in the revised manuscript.

**Results**

1. Could the numbers of pathogens detected by conventional testing and the study assay also be presented (rather than simply 'sole infection' and 'mixed infection')?

**Response:** As shown below, we have added in the Results section a detailed breakdown of pathogen detection frequencies. (Lines 273 to 282)

Sole infection caused by a single pathogen was observed in 58/66 (87.9%) of conventional test-positive samples, whereas real-time PCR detected a single pathogen in 30/124 (24.2%) of real-time PCR-positive samples. Mixed infections (two or more pathogens) were identified only in 8/66 (12.1%) of conventional test-positive samples but were substantially more frequent with real-time PCR, detected in 94/124 (75.8%) samples. When including pathogens not tested by conventional methods (*Y. enterocolitica*, *C. difficile*, STEC, and *D. fragilis*), the overall real-time PCR positivity was 131/153 (85.6%), with single infections in 37/131 (28.2%) and mixed infections in 94/131 (71.8%). The difference in infection patterns between these two methods was statistically significant (p<0.001).

2. Line 121-124 – this does not appear to be methods nor results; rather, it appears to be a conclusion of the authors, and so would be better placed in the Discussion

**Response:** We have moved this text to the Discussion section (Lines 325 to 329).

With few modifications this sentence was moved to the Discussion section**.** The high negative predictive value (>95%) observed for most pathogens indicates that the assay could serve as an effective screening tool to rule out infectious etiologies, potentially guiding appropriate antimicrobial therapy. Although the clinical efficacy of this assay requires further investigation, its application has the potential to improve the diagnosis of infectious gastroenteritis.

3. Line 162 – is this a description of how children are treated generally in the study setting or does it refer to the study population? If it refers to the study population, and is important, could it be made more specific?

**Response:** We have clarified this sentence as follows.

Among the enrolled pediatric patients, metronidazole (45.1%), ciprofloxacin (23.5%), and ofloxacin (23.5%) were the most commonly used antimicrobials. The other clinical characteristics are presented in **Table 1**. (Lines 266 to 268)

4. It seems curious that only 5% of the study population had fever. This probably deserves more comment – what was the chief complaint of the study population (ie. the reason they came to clinic or were hospitalized)? For example – were some of these children who were admitted with asthma exacerbation and just happened to have diarrhoea as well?

**Response:** In the revised manuscript, we have clarified the inclusion criteria and the clinical presentation as follows (Lines 135 to 137). Pediatric patients aged <5 years presenting diarrhea (with or without fever), defined as the passage of three or more loose or watery stools within a 24-hour period, were enrolled in the study. The predominance of watery diarrhea without fever suggests a viral or enterotoxigenic bacterial etiology, consistent with the high detection rates of norovirus, adenovirus, and ETEC in this cohort. We have also mentioned this point in the Discussion section as follows. (Lines 452 to 458)

The low prevalence of fever among the hospitalized patients reflects that the primary presentation is acute watery diarrhea without systemic signs of infection. Enrolled patients presented with moderate or severe diarrhea as their main symptom, and the inclusion criteria specifically required diarrhea as the primary cause for seeking medical care. The low fever rate is consistent with the high proportion of watery diarrhea rather than inflammatory or invasive diarrhea, which is more commonly associated with fever. Patients were not enrolled in this study if diarrhea was a secondary complaint or incidental finding.

5. Line 176-178 seems to be methods

**Response:** We have moved this methodological detail to the Methods section. (Lines 287 to 290)

In all real-time PCR-positive samples, an expected single melting peak has been observed with the corresponding pathogen and Tm values. Melting peaks were unambiguous; Tm distributions and replicate testing address potential borderline signals.

**Reviewer #2:-** Does the analysis presented match the analysis plan

Yes-partly

**Comments:** Are the results clearly and completely presented?

On line 88, authors indicate that Demographic and clinical information was also collected. However, only age and gender as well as clinical signs and symptoms (type of diarrhoea, and whether the patients had vomiting, fever, dehydration, or abdominal pain) were collected. This limited detail makes its slightly difficult to understand the study population better including demography, treatment, diagnosis, duration of illness or diarrhoea, outpatients versus inpatients, vaccination etc.

**Response:** We agree with the reviewer that additional demographic and clinical details would enhance the characterization of the study population. We have expanded this part in the Result section. (Lines 260 to 264)

A total of 153 diarrheal stool samples were collected from children below 5 years of age. The median age of the patients was 12 months (IQR 9,13) with almost an equal distribution of males (51.6%) and females (48.4%). Rotavirus universal vaccination program was initiated in our region in 2019. However, due to the COVID-19 pandemic, rotavirus vaccination could not be effectively implemented during the study period.

**Comment 2:** Performance, sensitivity, specificity, positive predictive value (PPV), negative predictive value (NPV), and likelihood ratio (LR) is indicated as the key focus of the study. However - only a paragraph attempts to discuss this - but in quite a scanty manner - lines 185 - 192. Also, the true data points are not presented on the text. for example - line 185 "Sensitivity for EPEC, Cryptosporidium, G. lamblia, and Rotavirus appeared low in this cohort, with wide confidence intervals". How can appearing low be judged? also line 188 - 189 "The specificity was good in detecting the majority of pathogens with fairly precise confidence intervals". Good?

**Response:** We acknowledge that the discussion of diagnostic performance metrics was insufficiently detailed. We have now expanded this section to provide specific values for each pathogen, interpret the clinical significance of these findings, and explain the criteria used for judgments (e.g., "low" sensitivity refers to values below 50% with confidence intervals crossing clinically unacceptable thresholds). We have also included the Likelihood Ratio (LR) data that was previously overlooked from the results presentation.

Sensitivity varied across pathogens. Sensitivity was 0% for EPEC (95%, CI: 0-84.2%) and *Cryptosporidium* spp. (95%, CI: 0-97.5%), 12.5% for *G. lamblia* (95%, CI: 0.3-52.7%), and 15% for rotavirus (95%, CI: 3.2-37.9), with wide confidence intervals due to a small number of positive samples (Table 3). In contrast, *Shigella*/EIEC demonstrated excellent sensitivity (100%, 95% CI: 69.2-100%) and high specificity (93%, 95% CI: 87.5-96.6%), resulting in greater diagnostic accuracy (93.5%). Norovirus also showed satisfactory performance with sensitivity of 87.5% (95%, CI: 47.3-99.7%) and specificity of 84.8% (95%, CI: 77.9-90.2%). The specificity exceeded 90% for several pathogens including *Shigella*/EIEC (93%), *Salmonella* spp. (92.1%), *Cryptosporidium* spp. (99.3%), *G. lamblia* (95.9%), and astrovirus (92.1%), with fairly precise confidence intervals (CI width ≤8%). The positive likelihood ratios (LR+) were greater than 10 for *Shigella/*EIEC (14.3), *Salmonella* spp (12.7), and astrovirus (12.7), indicating strong diagnostic utility, whereas negative likelihood ratios (LR-) were ≤0.3 for *Shigella*/EIEC, ETEC, *Salmonella* spp., norovirus, and astrovirus confirming the assay's utility as a screening tool. As shown in Table 3, the CUI based on NPV was high (>0.80) for *Shigella*/EIEC, *Salmonella,* *Cryptosporidium*, *G. lamblia*, norovirus, and astrovirus, indicating excellent negative clinical utility. Overall, diagnostic accuracy exceeded 90% for *Shigella*/EIEC (93.5%), *Salmonella* (92.2%), *Cryptosporidium* (98.7%), *G. lamblia* (91.5%), and astrovirus (92.2%). (Lines 291 to 308)

**Comment 3:** Are the figures (Tables, Images) of sufficient quality for clarity?

It's not clear to me why the melting peaks/curves are presented here (figure 2). I may be wrong, but I assumed that this would already have been validated by the manufacturer and therefore, only needed to be checked for validity including assessing positive and negative controls as part of standard operations expected of these experiments. This would be the same assumption I would easily make for the PCR amplification details highlighted in the methods. The expected melting curves and Tm ranges should already have been established by the manufacturer during assay development and validation - internal control, amplification controls and positive controls. Manufacturer establishes primer/probe design, analytical sensitivity/specificity, expected Tm windows, fluorescence channels, controls, compatible instruments and interpretation rules. The expectation here was checking that the assay performs as expected with the selected extraction method, thermocycler, stool samples, operators and laboratory conditions. Its likely that manufacturer provides software plus internal/positive/negative controls, but laboratories do review melting curves/Tm values for unusual, weak, multiple or borderline results.

**Response**: We appreciate this important clarification. The reviewer is correct that the manufacturer has established expected melting curves and Tm ranges during assay development. We have removed Figure 1 from the manuscript.

**Comment 4:** Figures: Both Figure 1 and 2 are not legible even when one zooms in. The coordinates and labels on both axis are not legible.

**Response:** We have removed the Figures from the manuscript.

**Other comments**

**Comment 5:** The median age of the study children can be reported in months considering the summary distributions - Age in years: Median (IQR) 1.0 (0.09, 1.11).

**Response:** We have revised the presentation of age to include both years and months for clarity.

The median age of the patients was 12 months (IQR 9,13) (Lines 260 to 261). This was also indicated in Table 1

**Comment 6:** The rates reported for conventional assays in line 164 - may need checking - detection rates were significantly higher with qPCR than in conventional testing (81% vs 43.8% - also in the abstract). I assumed its 41.8% i.e. 64/153 as indicated on table 2 - any pathogen positive sample by conventional method.

**Response:** Conventional detection rate was corrected as 66 (43.1%). We have included this information where ever necessary including in the revised Table 2.

**Abstract:**

‘Overall pathogen detection was significantly higher using this real-time PCR (81%) compared to the conventional assays (43.1%), reflecting differences in both sensitivity and pathogen panel breadth” (Lines 38 to 40)

**Results:**

**“**As shown in **Table 2**, detection rates were significantly higher with real-time PCR than in conventional testing (81% vs 43.1%), particularly for bacterial (61% vs 15.7%) and viral pathogens (67.3% vs 27%)”. (Lines 269 to 271)

**Comment 7:** Distribution of single and multiple infections needs to be reported for both conventional and PCR based methods

**Response:** We have now expanded the reporting of single and mixed infections to include specific frequencies and percentages for both conventional and real-time PCR methods.

Sole infection caused by a single pathogen was observed in 58/66 (87.9%) of conventional test-positive samples, whereas real-time PCR detected a single pathogen in 30/124 (24.2%) of real-time PCR-positive samples. Mixed infections (two or more pathogens) were identified only in 8/66 (12.1%) of conventional test-positive samples but were substantially more frequent with real-time PCR, detected in 94/124 (75.8%) samples. When including pathogens not tested by conventional methods (*Y. enterocolitica*, *C. difficile*, STEC, and *D. fragilis*), the overall real-time PCR positivity was 131/153 (85.6%), with single infections in 37/131 (28.2%) and mixed infections in 94/131 (71.8%). The difference in infection patterns between these two methods was statistically significant (p<0.001). (Lines 273 to 282)

**Comment 8:** Suggest that Table 4 be organised by types of pathogens similar to Table 3 for easy comparison.

**Response:** We have reorganized Table 4 to group pathogens by type (Bacteria, Parasites, Viruses) to maintain consistency with Table 3.

**Comment 9:** Several wording and tense phrases need checking e.g. line 176 - have been detected very frequently, line 181 - has been observed. Check elsewhere.

**Response:** We have carefully reviewed the entire manuscript for grammatical errors, tense inconsistencies, and complex phrasing. All corrections have been made throughout the document.

**Conclusions**

**Comment 10**: I think the Discussion section could do with reworking to better place the results in the context of other studies and to better expand on the limitations of the study.

**Response:** We have substantially restructured the Discussion section under five subheadings as shown below. We have also included limitations of this study.

Enhanced pathogen detection and clinical utility of the GastroFinder 2SMART assay

Geographical variations of common enteric pathogens

Diagnostic performance metrics across pathogen types

Comparison of frequentist and Bayesian analytic approaches

Feasible considerations for LMIC settings

**Comment 11:** The first three paragraphs of the discussion appears to be background information. I would recommend using the beginning of the discussion to discuss the most important results of the study.

**Response:** We have restructured the Discussion section. The revised version covers the most important findings, including contextualization with geographical variations.

**Comment 12:** Line 229 – underdiagnosis of rotavirus would be a fatal flaw for an assay designed for paediatrics, given the morbidity and mortality caused by this pathogen in many parts of the world. I appreciate the authors' having thought about WHY this might have occurred – but the impact of this deficiency needs to also be discussed in detail.

**Response:** We have expanded our discussion of the rotavirus underdiagnosis issue to more fully address its clinical implications (Lines 330 to 351).

The low rotavirus detection rate by the real-time PCR assay represents a critical limitation in this study. Rotavirus remains one of the leading causes of severe dehydrating diarrhea in children under 5 years globally, accounting for approximately 39% of diarrhea-related hospitalizations and 28% of deaths in this age group [1]. In India, rotavirus is estimated to cause approximately 78,000 deaths annually among children under five years of age, and has been a priority target for the National Immunization Program since 2016 [<https://sansad.in/getFile/loksabhaquestions/annex/8/AU1082.pdf?source=pqals> Accessed on July 11, 2026]. The substantially lower sensitivity of this assay for rotavirus compared to conventional RT-PCR (which targets VP6) suggests that the NSP3 gene target used in the real-time PCR assay may be suboptimal for clinical detection. NSP3 is a non-structural protein expressed at lower levels during infection compared to the structural VP6 protein, which may result in lower detection sensitivity, particularly when multiple targets are multiplexed [16]. Furthermore, the higher Tm values observed (62-65) indicate low target abundance or potential competition for reagents in the multiplex reaction.

Clinical implications of this underdiagnosis are significant. False-negative rotavirus results could lead to: (1) inappropriate antibiotic use to control diarrhea, contributing to antimicrobial resistance; (2) failure to implement appropriate infection control measures, increasing transmission risk in healthcare settings; (3) missed opportunities for rotavirus vaccine efficacy monitoring; and (4) underestimation of the true rotavirus disease burden in community and hospital settings. For these reasons, clinicians should consider supplementing this assay with a VP6-targeting test (either ELISA or RT-PCR) when clinical suspicion of rotavirus is high, or seek alternative multiplex assays that include robust VP6-targeting primers.

**Comment 13**: Line 239 – this paragraph is also mostly background

**Response:** We have deleted this sentence.

**Comment 14:** Line 246 – I think the intent of this paragraph is to compare the relative prevalence of certain pathogens in the study population with that in different areas. If so, it should be rewritten to more clearly compare and contrast so that the intended conclusions can be drawn...

**Response:** We have rephrased this paragraph to compare pathogen distribution in our study with findings from other geographical regions. (Lines 353 to 364)

The distribution of enteric pathogens in our study population showed both similarities and notable differences compared to other geographical regions. Norovirus and adenovirus (20.3%) were the most frequently detected viral pathogens, which is consistent with findings from recent studies in India [11] and other South Asian countries [17]. However, the rotavirus detection rate (3.9%) was substantially lower than the 25-40% typically reported in the pre-vaccination period [18], reflecting the assay limitations. Among bacterial pathogens, *Campylobacter* spp. and *Shigella*/EIEC were predominant, similar to findings from MAL-ED cohort sites in Bangladesh and Pakistan [2]. In contrast, studies from African sites (Côte d'Ivoire and Mali) have reported higher prevalence of EAEC and *Campylobacter* [19], while *Salmonella* was more commonly detected in Southeast Asian settings [20]. These geographical variations highlight the importance of region-specific diagnostic panels and the need for local epidemiological data to inform clinical practice.

**REVIEWER #2**

**Comment 15:** Overall, the conclusions don't follow from the results and are broadly general and need careful rewording.

**Response:** We have modified the Conclusion section to be more specific, evidence-based, and derived from our results. (Lines 486 to 500)

The GastroFinder 2SMART multiplex real-time PCR assay demonstrates significant utility for enhancing pathogen detection in pediatric diarrheal stools, with an overall detection rate 2-fold higher than conventional methods. The assay shows excellent diagnostic performance for certain pathogens, supporting its use in clinical settings. The high NPV observed for most pathogens suggests the assay could serve as an effective screening tool to rule out infectious etiologies, potentially reducing unnecessary antibiotic use. However, the assay has critical limitations, most notably the underdiagnosis of rotavirus, which precludes its use as a stand-alone diagnostic for rotavirus-associated diarrhea. Additionally, the assay's ability to detect nucleic acids from both viable and non-viable organisms may complicate clinical interpretation, particularly in settings where asymptomatic carriage is common. When used judiciously, this molecular approach has the potential to improve patient management, support antimicrobial stewardship, and enhance surveillance of diarrheal disease epidemiology. Future studies should evaluate the cost-effectiveness of this assay in LMIC settings and its impact on clinical outcomes.

**Comment 16:** The limitations described are few and could easily be expanded.

**Response:** We have expanded the limitations section to provide a comprehensive account of the study's constraints. (Lines 462 to 485)

This study has several important limitations that should be considered when interpreting the findings. First, the sample size was comparatively small, particularly for evaluating rare pathogens, resulting in wide confidence intervals for several estimates. Second, conventional methods were used as the reference standard for diagnostic accuracy calculations, but these methods are inadequate and may underestimate true pathogen prevalence. While we addressed this using Bayesian latent class analysis, which accounts for weak reference standards, the conditional independence assumption may not fully hold for all pathogens. Third, we did not perform orthogonal confirmation for all real-time PCR-positive samples. Fourth, the tested real-time PCR does not relate pathogen-load thresholds for defining positivity; therefore, some mixed infections may represent low-level detections of colonizing organisms rather than true active infections, potentially overestimating the polymicrobial infection. Fifth, the study was conducted in a single geographic region, which may limit generalizability to other settings with different pathogen distributions and epidemiological characteristics. Sixth, we did not systematically evaluate the cost-effectiveness of the assay compared to conventional methods, which is a critical consideration for implementation in resource-limited settings. Seventh, vaccination status was not available for all participants due to the COVID-19 pandemic, limiting our ability to assess the impact of rotavirus vaccination on detection rates. Eighth, the clinical relevance of detecting multiple pathogens remains uncertain, as it is unclear whether all detected organisms are contributing to disease. Finally, because the conventional diagnostic algorithm reflected routine clinical practice in our region, direct comparisons could only be performed for pathogens routinely investigated by conventional laboratory methods, which may limit the comprehensiveness of our performance evaluations for less commonly tested organisms.

**Comment 17:** The first paragraph needs a summary of the results and not background details which are covered in the introduction. Line 200 - 226 appear mostly background details. The discussion also lacks coherence and would have benefited if an initial structure was developed and followed to avoid randomness.

**Response:** We have restructured the entire Discussion section to follow a logical, coherent structure as shown below

Enhanced pathogen detection and clinical utility of the GastroFinder 2SMART assay

Geographical variations of common enteric pathogens

Diagnostic performance metrics across pathogen types

Comparison of frequentist and Bayesian analytic approaches

Feasible considerations for LMIC settings

**Comment 18:** The focus of the study results has not been discussed - the study evaluated GastroFinder® 2SMART kit to check its performance, sensitivity, specificity, positive predictive value (PPV), negative predictive value (NPV), and likelihood ratio (LR) - line 75 - 77. Diagnostic performance measures including sensitivity, specificity, predictive values, accuracy, and probabilistic clinical utility index (CUI) were estimated with 95% confidence intervals (CI) - line 129 - 131. However, none of these is available in the discussion section. The results from the study are actually rarely discussed or interpreted to get meaningful insight what this truly means. There is a bit of detail on Rotavirus but not the other pathogens or pathogen groups.

**Response:** We have added a section to the Discussion that interprets the diagnostic performance metrics for all pathogen groups and provides clinical context for the findings. (Lines 365 to 405)

**Diagnostic performance metrics across pathogen types**

Since the conventional diagnostic algorithm reflected routine clinical practice in our region, direct comparisons could only be performed for pathogens routinely investigated by existing laboratory methods. In this study, the diagnostic performance of the tested real-time PCR assay varied substantially across pathogen types. For bacterial pathogens, the assay demonstrated excellent sensitivity for *Shigella*/EIEC (100% vs conventional, 93.2% by LCM) and moderate sensitivity for *Campylobacter* (50% vs conventional, 51.3% by LCM) and ETEC (100% vs conventional, 52.6% by LCM). These findings align with previous evaluations of multiplex gastrointestinal panels, which have reported sensitivities ranging from 89-100% for bacterial pathogens [21, 22]. The high specificity (>90%) and positive likelihood ratios (>10) for *Shigella/*EIEC indicate that a positive result effectively confirms infection, with strong diagnostic utility for clinical decision-making. The excellent NPV (>97%) for all bacterial pathogens suggests that negative results effectively rule out these infections, supporting the assay's use as a screening tool. The detection of pathogens not routinely tested in conventional panels (*Y. enterocolitica*, *C. difficile*, STEC, and *D. fragilis*) adds value to the assay, as these organisms are often overlooked despite their clinical significance.

For viral pathogens, norovirus showed favorable performance (sensitivity 87.5%, specificity 84.8%), which is consistent with the known utility of molecular methods [2]. The moderate performance for adenovirus (sensitivity 78.6%, specificity 56.1%) is similar to previous reports of reduced specificity in multiplex panels due to asymptomatic carriage [21]. The poor sensitivity for rotavirus (15%) and sapovirus (0%) highlights the need for alternative or supplementary tests for these pathogens [23]. For parasitic pathogens, specificity was excellent (>95%) for all parasites, indicating that positive results are reliable. However, sensitivity was poor for *Cryptosporidium* spp. (0%) and *G. lamblia* (12.5%), likely reflecting the high parasite load required for detection in multiplex reactions and competition for reagents. These findings suggest that dedicated parasitological methods like microscopy or single-target PCR remain necessary for parasite diagnosis in clinical settings.

The discordance between conventional and molecular methods warrants careful consideration. Only 10% of intestinal bacteria can be cultured using selective media [24]. For bacterial pathogens, low culture detection rates might reflect several factors: selective media may not support the growth of all strains. Molecular methods on the other hand detect both viable and non-viable organisms, and the detection rate is twofold higher than culture methods [25]. The moderate concordance observed for some viral pathogens is concerning and may be attributed to target mismatch between assays. Conventional viral methods used simplex/multiplex PCR targeting different gene regions than the real-time PCR test, which could explain some discordance. Additionally, fecal specimens contain residual inhibitors such as heme, bilirubin, bile salts, and complex carbohydrates that reduce PCR efficiency by interacting with target DNA/RNA or inactivating Taq polymerases [26]. These findings underscore that conventional methods are inadequate reference standards. While Bayesian models rely on conditional independence assumptions, other robust validation strategies can identify the limitations and ensure reliable estimates [27].

**Comment 19:** There is a drive towards pushing GastroFinder 2SMART test as a molecular point-of-care testing but this possibly needs some thoughts on cost implications, feasibility, diagnostic utility etc considering the results described here - sensitivity for some of the well-known diarrheal pathogens was low - while details of certain pathogens in the population are not provided e.g. Yersinia, C. diff., STEC, etc. Could the authors highlight "the diagnostic usefulness" of the assay especially for a LMIC settings and implications for mixed infections. For this assay, what would over/underdiagnosis look like in field scenarios considering the different stages of disease and patient journeys seeking healthcare?

**Response:** We have added a section addressing the practical implications for LMIC settings, including cost, feasibility, and the clinical significance of mixed infections. (Lines 436 to 461)

**Feasible considerations for LMIC settings**

The application of multiplex molecular diagnostics in resource-limited settings requires careful consideration of cost, infrastructure, and clinical utility. The GastroFinder 2SMART assay requires a real-time PCR thermocycler, trained laboratory personnel, and reliable electricity and cold chain for reagent storage—resources that may not be available in peripheral healthcare facilities in many LMIC settings. The estimated cost per test may be substantially higher than conventional methods but less comparable to other commercial multiplex panels.

In field scenarios, the high NPV of the assay (>95% for most pathogens) could support a "rule-out" strategy, where negative results guide clinicians to consider non-infectious causes of diarrhea and avoid unnecessary antibiotics. However, the low sensitivity for rotavirus and parasites means that negative results do not reliably exclude these pathogens, potentially leading to underdiagnosis. The high rate of mixed infections detected raises important questions about clinical management: it remains unclear whether all detected pathogens are contributing to disease, and whether treatment should target all or only the most clinically significant pathogens. In the absence of quantitative thresholds, clinicians may face diagnostic uncertainty.

The low prevalence of fever among the hospitalized patients reflects that the primary presentation is acute watery diarrhea without systemic signs of infection. Enrolled patients presented with moderate or severe diarrhea as their main symptom, and the inclusion criteria specifically required diarrhea as the primary cause for seeking medical care. The low fever rate is consistent with the high proportion of watery diarrhea rather than inflammatory or invasive diarrhea, which is more commonly associated with fever. Patients were not enrolled in this study if diarrhea was a secondary complaint or incidental finding. This clinical context is important for LMIC settings where syndromic management is common, as it underscores the need for accurate diagnostics to differentiate viral from bacterial etiologies and avoid unnecessary antibiotic use.

**Comment 20:** The concordance or discrepance of the frequentists versus the Bayesian latent class analysis on performance metrics needs to be described or compared.

**Response:** We have added a comparison of the frequentist (conventional reference standard) and Bayesian latent class analysis results to the Discussion section. (Lines 406 to 435)

**Comparison of frequentist and Bayesian analytic approaches**

The Bayesian latent class analysis should be interpreted in the context of its underlying assumptions. Although we assumed conditional independence between the conventional methods and multiplex real-time PCR, given the true infection status, this assumption may not be adequate in routine clinical practice. Both these methods are influenced by pathogen burden, while culture-based methods may additionally be affected by bacterial viability and recent antimicrobial exposure. The Bayesian estimates remain dependent on model assumptions and should be interpreted cautiously, particularly for low prevalence pathogens.

We observed substantial concordance between the frequentist estimates (using conventional methods as the reference standard) and Bayesian latent class analysis estimates for most pathogens. For *Shigella*/EIEC, both approaches yielded excellent sensitivity and specificity, although Bayesian estimates were associated with wider uncertainty. Similarly, Norovirus estimates were consistent, indicating robust detection across analytic methods. The Youden Index from Bayesian analysis ranked *Shigella*/EIEC highest, corroborating conventional accuracy metrics.

However, notable discrepancies were observed for pathogens with low prevalence in the study population. For *Salmonella* spp., astrovirus, and *Campylobacter*, Bayesian estimates of sensitivity were lower than frequentist estimates, with wide credible intervals reflecting uncertainty due to few positive samples. These findings indicate the value of the latent class approach in situations where the reference standard is imperfect, as it provides complementary estimates by accounting for the limitations of both testing methods.

The discordance between approaches was most pronounced for pathogens where conventional methods are known to have low sensitivity. In these cases, the frequentist approach may underestimate true sensitivity, while the Bayesian approach provides a more conservative but likely more accurate estimate. For clinical decision-making, we endorse using the Bayesian provides alternative estimates for pathogens where conventional methods are unreliable, but caution that the wide credible intervals for low-prevalence pathogens limit their interpretative value. Because the conventional diagnostic algorithm reflected routine clinical practice in our region, direct comparisons could only be performed for pathogens routinely investigated by conventional laboratory methods.

**Response to summary and general comments**

**Reviewer #1:** The authors should be commended for evaluating another multiplex assay for enteric pathogen detection, given the morbidity and mortality attributable to acute diarrhoeal disease in South Asia. However, I think major revisions to this article would be required to consider it for publication in this journal. I am unsure of exactly how much better the assay is than conventional testing given the lack of detail provided on what was 'conventional' in this study population and the lack of description of the study population; I am also unfamiliar with some of the metrics used to measure assay performance. Data availability seems very vague.

**Response:** We thank the reviewer for the positive recognition of the study's importance. We have addressed all concerns as detailed above. Specifically, we have:

1. Provided detailed descriptions of conventional methods (bacterial culture, RT-PCR for viruses, microscopy for parasites) in the Materials and Methods section

2. Expanded the study population description with demographic and clinical details

3. Explained all performance metrics (CUI, likelihood ratios, Youden Index) with clear definitions

Data Availability Statement has been included in the revised manuscript.

The datasets generated and/or analyzed during the current study are available from the corresponding authors upon reasonable request. All data supporting the findings is included within the manuscript and its supplementary files.

**Reviewer #2:** The study by Chowdhury and colleagues is an important one in attempting to enumerate the etiology of diarrhoea infections in young children which are linked to mortality and morbidity in many LMIC settings. They focussed on a customised commercially produced PCR based assay targeting 17 enteric pathogens (bacteria, virus, and parasites) which are players in diarrhoea aetiology. The overall outlook of the paper is that it needs quite some strengthening across the methods, results and discussion to help with interpretation.

**Response:** We thank the reviewer for recognizing the importance of our study. We have carefully revised the manuscript to strengthen all sections, improve interpretation of results, and provide clearer guidance on the clinical utility of the assay, particularly in LMIC settings.
