# Supplementary material for: Enhancing pathogen detection in pediatric diarrheal stools: The case of molecular multiplexing with a real-time PCR assay
Source: PLoS Negl Trop Dis. 2026 Nov 2;20(11):e0014823. doi: 10.1371/journal.pntd.0014823 (PMC13750648; doi:10.1371/journal.pntd.0014823)
Supplement: Supplementary file 3 [file pntd.0014823.s004.docx]

**Response to Reviewers' Comments**

**Manuscript Title:** Enhancing Pathogen Detection in Pediatric Diarrheal Stools: The Case of Molecular Multiplexing with a Real-time PCR Assay

**Journal:** *PLOS Neglected Tropical Diseases*

**Manuscript ID:** PNTD-D-26-00570R1

We sincerely thank the Academic Editors and the Reviewers for their constructive comments and suggestions, which have helped significantly improve the quality and clarity of our manuscript. Below, we provide a point-by-point response to all comments raised by the reviewers.

**Response to Reviewer 1**

**Comment:** Exclusion criteria – you do not need to specify criteria that are the inverse of inclusion criteria…just add the exclusions that are applicable AFTER following inclusion criteria.

**Response:** We agree with the reviewer and have streamlined the Exclusion Criteria in the Methods section by removing statements that inverted the inclusion criteria. The section now focuses strictly on criteria applied after initial inclusion screening.

**Revised text (Methods):**

"**Exclusion criteria**: Children with a history of antibiotic use prior to stool sample collection, those without diarrhea, and children presenting with additional non-diarrheal diagnoses as the primary admission cause. Although children with a history of antibiotic use prior to stool sample collection were excluded, undisclosed antibiotic use cannot be ruled out. Stool samples that were inadequate in quantity, improperly collected, or not processed within the recommended time were also excluded. (Lines 143-148)

**Comment:** Would specify how many stools were collected and then how many were excluded (because of sample quality, previous antibiotic use, etc) to get to the final number of 153.

**Response:** During the study period, a total of 153 pediatric stool specimens were collected from patients presenting with acute diarrhea. As clinicians strictly evaluated and followed the sample collection criteria prior to sending the specimens to the laboratory, no samples required exclusion, and all 153 specimens were included in the final diagnostic evaluation. The above information has been included in the rerevised version of the manuscript (Lines 149-152).

**Comment:** I am still not sure that the CUI values add anything beyond what one would understand looking at sensitivity, specificity, PPV, and NPV... Is there a reference for the definition of CUI for diagnostic tests? Is there a reference for the grading (excellent-good-fair-poor-very poor) of the CUI? In contrast, I do not think sensitivity, specificity, PPV, and NPV need to be defined (lines 224-229), as these are very common terms.

**Response:** We appreciate this critique. We have removed the definitions of sensitivity, specificity, PPV, and NPV. We retained Clinical Utility Index (CUI) as a complementary summary measure integrating diagnostic accuracy and predictive value to reflect the clinical utility of positive and negative test results and added the appropriate reference for its calculation and qualitative grading. A new reference has been included in the text (Mitchell, 2011).

**Revised text (Methods):**

The clinical utility index (CUI) provides a measure of the practical usefulness of a diagnostic test in clinical practice (Mitchell, 2011). CUI combines test accuracy with prevalence information and is calculated as: CUI+ = Sensitivity × PPV for positive tests, and CUI- = Specificity × NPV for negative tests. Grading thresholds for CUI follow established conventions: >0.81 excellent, 0.64–0.80 good, 0.49–0.63 fair, 0.36–0.48 poor, and <0.36 very poor (Mitchell, 2011). (Lines 214-219).

Reference: Mitchell AJ. Sensitivity × PPV is a recognized test called the clinical utility index (CUI+). Eur J Epidemiol. 2011;26(3):251-2; author reply 252. doi: 10.1007/s10654-011-9561-x.

**Comment:** Lines 262-264 – this information about rotavirus vaccine and uptake could be included with the rest of the description of the setting in the Methods.

**Response:** The sentences describing the rotavirus national immunization program and the COVID-19 pandemic-related disruption have been moved from the Results section to the Methods section.

**Revised text (Methods):**

The rotavirus universal vaccination program was initiated in our region in 2019; however, due to the COVID-19 pandemic, rotavirus vaccination could not be effectively implemented during the study period (Lines 123-125).

**Comment:** There were 25 STEC detected?? I find that very surprising that there were more STEC than other pathogens that I would have thought to be much more common (including Salmonella, Shigella, rotavirus, EPEC). Why was this not included in the table? One probably should not say that they are ‘infrequently encountered’ if they are frequently encountered…

**Response:** We thank the reviewer for pointing out this subject. STEC was detected in 25 samples by GastroFinder 2SMART, which was unexpected in our region. STEC and other non-panel targets were excluded from head-to-head performance comparisons in Table 3 because conventional culture for STEC (e.g., sorbitol-MacConkey agar or *stx1/stx2* PCR) was not part of our routine laboratory algorithm. We have corrected the wording regarding "infrequently encountered" and added a separate paragraph in the Discussion evaluating STEC detection.

**Revised text:**

Results section: "Notably, GastroFinder 2SMART detected Shiga toxin-producing *E. coli* (STEC) in 25 samples (16.3%). STEC was not included in our routine conventional panel due to its rare representation in clinical samples from our region, and thus excluded from head-to-head performance comparisons”. (Lines 277-280).

Discussion section: “Molecular detection of STEC highlights an under-recognized pathogen burden in pediatric diarrhea in Eastern India. However, because molecular panels detect toxin genes without differentiating active infection from low-level colonization, quantitative load assessment and local epidemiological validation remain necessary." (Lines 334-337)

**Comment:** I still am not sure how the Youden Index helps the reader understand here. The Youden Index will be optimized when sensitivity and specificity are both high…but since the ‘probable true’ sensitivity of the reference is low, specificity for multiple targets calculates to be much lower than it should be in ‘reality’. Wouldn’t it be easier just to present McNemar P values showing that the 2SMART and conventional don’t agree for very many targets?

**Response:** We acknowledge the reviewer's comment. We agree that imperfect reference standard may affect sensitivity and specificity. The Youden index provides a complementary summary of diagnostic discrimination, whereas McNemar's test assesses differences in paired positivity rates. As these addresses different statistical questions, we retained Youden Index. We have included McNemar’s test *p*-values in Table 3.

**Comment:** Line 317: Is the main thrust of the manuscript that the 2SMART assay is much better than conventional methods or that real-time PCR is better than conventional methods? This should be clarified... the 2SMART has serious limitations for use in children in LMICs if it cannot detect rotavirus properly... it probably deserves to also be included in the first sentence of the discussion.

**Response:** We have revised the opening paragraph of the Discussion to explicitly state that while multiplex real-time PCR substantially enhances overall pathogen detection over conventional culture, specific commercial panels like GastroFinder 2SMART exhibit crucial limitations—most notably critical rotavirus underdiagnosis—which impact their utility as standalone pediatric tools in LMICs.

**Revised text (Discussion):**

"This study demonstrates that multiplex real-time PCR significantly enhances overall pathogen detection in pediatric diarrheal stools compared to conventional methods. However, the evaluation highlights a critical diagnostic weakness in the GastroFinder 2SMART assay: a severe lack of sensitivity for rotavirus, which limits its standalone utility in pediatric populations where rotavirus is a leading pathogen." (Lines 315-319).

**Comment:** Line 325 – whether the testing improves antimicrobial therapy use is dependent on a number of different factors, including: timing of testing; whether antimicrobials are used at admission for diarrhoea or for other illnesses; whether clinicians would be receptive to changing therapy based on test results; availability of different antibiotics; etc.

**Response:** We agree and have modified the text in the Discussion to reflect that real-time PCR testing can support antimicrobial stewardship only when integrated into rapid clinical workflows, alongside clinician receptivity and rapid turnaround time.

**Revised text (Discussion):**

"The high negative predictive value (>95%) observed for most pathogens indicates that the assay could serve as an effective screening tool to rule out infectious etiologies. However, translating these diagnostic gains into improved antimicrobial stewardship depends on several operational factors, including rapid test turnaround times, sampling prior to empiric administration, clinician willingness to de-escalate therapy based on negative molecular results, and institutional antibiotic guidelines (Brendish et al., 2023)." (Lines 327-332)

Reference: Brendish NJ, Beard KR, Malachira AK, et al. Clinical impact of syndromic molecular point-of-care testing for gastrointestinal pathogens in adults hospitalised with suspected gastroenteritis (GastroPOC): a pragmatic, open-label, randomised controlled trial. Lancet Infect Dis. 2023;23(8):945-955. doi: 10.1016/S1473-3099(23)00066-X.

**Comment:** Line 333 – this reference should probably be replaced by the updated GBD paper published in Lancet ID in June 2026.

**Response:** We have updated the reference [2] to the latest Global Burden of Disease Diarrhoeal Diseases Collaborators study published in *Lancet Infectious Diseases* (GBD Diarrhoeal Diseases Collaborators, 2026).

Reference # 2: GBD 2023 Diarrhoeal Disease and Enteric Infectious Diseases Collaborators. Global burden of enteric infectious diseases, diarrhoeal diseases, and corresponding aetiologies, 1990-2023: a systematic analysis for the Global Burden of Disease Study 2023. Lancet Infect Dis. 2026; S1473-3099(26)00194-5.

**Comment:** Line 381 – the sensitivity of 87% of the 2SMART for noro detection compared to conventional methods does not seem that good.

**Response:** We have revised our interpretation of the 87.5% norovirus sensitivity, subduing our tone to acknowledge that while superior to conventional RT-PCR in total yield, an 87.5% sensitivity leaves room for missed cases during peak viral outbreaks.

**Revised text (Discussion):**

"For viral pathogens, norovirus showed moderate sensitivity (sensitivity 87.5%, specificity 84.8%) relative to single-target RT-PCR, indicating that while yield is improved, a subset of norovirus infections may still escape multiplex detection due to competitive inhibition between different primers that sometimes reduce overall diagnostic sensitivity compared to simplex RT-qPCR assays (Kulis-Horn et al., 2020)." (Lines 390-394).

Reference: Kulis-Horn RK, Tiemann C. Evaluation of a laboratory-developed test for simultaneous detection of norovirus and rotavirus by real-time RT-PCR on the Panther Fusion® system. Eur J Clin Microbiol Infect Dis. 2020 Jan;39(1):103-112. doi: 10.1007/s10096-019-03697-7.

**Comment:** Line 384 – why would poor specificity of the adeno target in THIS cohort be due to asymptomatic carriage? The underlying assumption in this study was that all had infectious diarrhoeal disease.

**Response:** We thank the reviewer for this observation. Since all children in this study presented with symptomatic diarrhea, low specificity relative to culture/conventional PCR reflects molecular detection of low viral shedding, low-level co-infections, or prolonged fecal excretion from a prior episode rather than asymptomatic healthy carriage. We have corrected this phrasing in the Discussion.

**Revised text (Discussion):**

"The moderate performance for adenovirus (sensitivity 78.6%, specificity 56.1%) is similar to previous reports of reduced specificity in multiplex panels, wherein symptomatic pediatric cohorts primarily reflect prolonged viral shedding from past infections or low target copy numbers detected by PCR but below the detection threshold of conventional assays (Leli et al., 2020)." (Lines 394-398).

**Comment:** Line 438 – is the 2SMART harder to stand up in an LMIC setting than a micro lab capable of doing culture and other traditional techniques (eg TSI)?

**Response:** We have expanded the discussion on LMIC implementation to compare the practical challenges of running a superior molecular platform versus traditional microbiology laboratories.

**Revised text (Discussion):**

"While conventional microbiology requires manual effort and specialized expertise, its basic equipment infrastructure is widely available and low-cost. Conversely, implementing multiplex molecular assays like GastroFinder 2SMART requires substantial upfront capital for multi-channel real-time PCR thermocyclers, uninterrupted power supply, continuous cold-chain infrastructure, and expensive reagent kits. Thus, in many LMIC settings, molecular panels are currently more feasible as centralized referral diagnostics rather than point-of-care tools." (Lines 449-455).

**Comment:** Line 453 – it is stated ‘enrolled patients presented with diarrhoea as their main symptom.’ How is this known? The eligibility criteria do not state anything about excluding children who had additional non-diarrhoeal diagnoses or whose main reason for admission to hospital was not diarrhoea. If that IS the case – the eligibility criteria should be revised.

**Response:** We have updated the Inclusion and Exclusion criteria under Methods to explicitly specify that diarrhea was the primary reason for hospital presentation/admission.

**Revised text (Methods):** (Lines 138-148)

**Inclusion criteria:**  Pediatric patients aged <5 years presenting diarrhea (with or without fever), defined as the passage of three or more loose or watery stools within a 24-hour period, where diarrhea was the primary reason for seeking medical care, were enrolled in the study. Stool samples were collected after obtaining written informed consent from the parent or legal guardian and before the initiation of antibiotic therapy.

**Exclusion criteria:**  Children with a history of antibiotic use prior to stool sample collection, those without diarrhea, and children presenting with additional non-diarrheal diagnoses as the primary admission cause. Although children with a history of antibiotic use prior to stool sample collection were excluded, undisclosed antibiotic use cannot be ruled out. Stool samples that were inadequate in quantity, improperly collected, or not processed within the recommended time were also excluded.

**Comment:**

- The first line of the abstract is still incorrect. Diarrhoea is not an infectious disease by definition (many noninfectious pathologies can cause diarrhoea).
- Line 92 – the URL should probably be placed in the reference list.
- Line 106 – consider using ‘high-income’ instead of ‘developed’ countries.

**Response:** All three editorial points have been corrected:

1. Abstract opening sentence corrected to: "Diarrhea is a leading cause of pediatric morbidity and mortality worldwide, frequently caused by various bacterial, viral, and protozoan pathogens." (Lines 32-33).
2. The WHO factsheet URL has been moved from the text into the formal reference list (WHO, 2024).
3. "developed countries" changed to "high-income countries". (Line 107)

**Response to Reviewer #2**

**Comment:** I find the word “significant” - line 487 and “excellent” line 489 to be somewhat an overstatement based on the assay limitations. This language can be moderated in the final versions.

**Response:** We have moderated the language in the Conclusions and Abstract, replacing hyperbolic terms like "significant utility" and "excellent performance" with objective, nuanced descriptions acknowledging both the increased detection yield and the diagnostic limitations (e.g., rotavirus underdiagnosis).

**Revised text (Conclusion):**

"The GastroFinder 2SMART multiplex real-time PCR assay demonstrates substantial analytical capacity for enhancing pathogen detection in pediatric diarrheal stools, with an overall detection rate 2-fold higher than conventional methods. The assay shows high diagnostic sensitivity for selected bacterial targets like *Shigella*/EIEC, supporting its use in clinical settings. However, the assay has critical limitations, most notably the severe underdiagnosis of rotavirus…….." (Lines 502-507).

**Comment:** The revised version has greatly improved the manuscript - the discussion sections can have the subtitles withdrawn but leave limitations and conclusions subheadings.

**Response:** In accordance with the reviewer's recommendation, we have removed the thematic subheadings within the main body of the Discussion while retaining the dedicated "Limitations" and "Conclusions" section headings.
