# Supplementary material for: Enhancing pathogen detection in pediatric diarrheal stools: The case of molecular multiplexing with a real-time PCR assay
Source: PLoS Negl Trop Dis. 2026 Nov 2;20(11):e0014823. doi: 10.1371/journal.pntd.0014823 (PMC13750648; doi:10.1371/journal.pntd.0014823)
Supplement: Peer review history

# Peer review: Decision Letter 0

**DOI:** 10.1371/journal.pntd.0014823.r001

**Authors:** Mathieu Picardeau, Kirkby Tickell

23 Jun 2026

PNTD-D-26-00570

Enhancing Pathogen Detection in Pediatric Diarrheal Stools: The Case of Molecular Multiplexing with a Real-time PCR (qPCR) Assay

PLOS Neglected Tropical Diseases

Dear Dr. Ramamurthy,

Thank you for submitting your manuscript to PLOS Neglected Tropical Diseases. After careful consideration, we feel that it has merit but does not fully meet PLOS Neglected Tropical Diseases's publication criteria as it currently stands. Therefore, we invite you to submit a revised version of the manuscript that addresses the points raised during the review process.

Please submit your revised manuscript within by Aug 22 2026 11:59PM. If you will need more time than this to complete your revisions, please reply to this message or contact the journal office at plosntds@plos.org. When you're ready to submit your revision, log on to https://www.editorialmanager.com/pntd/ and select the 'Submissions Needing Revision' folder to locate your manuscript file.

Please include the following items when submitting your revised manuscript:

* A letter that responds to each point raised by the editor and reviewer(s). You should upload this letter as a separate file labeled 'Response to Reviewers'. This file does not need to include responses to any formatting updates and technical items listed in the 'Journal Requirements' section below.

* A marked-up copy of your manuscript that highlights changes made to the original version. You should upload this as a separate file labeled 'Revised Manuscript with Track Changes'.

* An unmarked version of your revised paper without tracked changes. You should upload this as a separate file labeled 'Manuscript'.

If you would like to make changes to your financial disclosure, competing interests statement, or data availability statement, please make these updates within the submission form at the time of resubmission. Guidelines for resubmitting your figure files are available below the reviewer comments at the end of this letter.

As the corresponding author, your ORCID iD is verified in the submission system and will appear in the published article. PLOS supports the use of ORCID, and we encourage all coauthors to register for an ORCID iD and use it as well. Please encourage your coauthors to verify their ORCID iD within the submission system before final acceptance, as unverified ORCID iDs will not appear in the published article. Only the individual author can complete the verification step; PLOS staff cannot verify ORCID iDs on behalf of authors.

We look forward to receiving your revised manuscript.

Kind regards,

Kirkby D Tickell, MBBS BSc

Academic Editor

PLOS Neglected Tropical Diseases

Mathieu Picardeau

Section Editor

PLOS Neglected Tropical Diseases

Shaden Kamhawi

co-Editor-in-Chief

PLOS Neglected Tropical Diseases

orcid.org/0000-0003-4304-636XX

Paul Brindley

co-Editor-in-Chief

PLOS Neglected Tropical Diseases

orcid.org/0000-0003-1765-0002

**Additional Editor Comments (if provided):**

**Journal Requirements:**

If the reviewer comments include a recommendation to cite specific previously published works, please review and evaluate these publications to determine whether they are relevant and should be cited. There is no requirement to cite these works unless the editor has indicated otherwise.

1) Please ensure that the CRediT author contributions listed for every co-author are completed accurately and in full.

At this stage, the following Authors/Authors require contributions: Goutam Chowdhury, Melissa Glenda Lewis, Alok Deb, Mamta Chawla-Sarkar, Sandipan Ganguly, Paulami Dutta, Santasabuj Das, Asish K. Mukhopadhyay, and Thandavarayan Ramamurthy. Please ensure that the full contributions of each author are acknowledged in the "Add/Edit/Remove Authors" section of our submission form.

The list of CRediT author contributions may be found here: https://journals.plos.org/plosntds/s/authorship#loc-author-contributions

2) Please provide an Author Summary. This should appear in your manuscript between the Abstract (if applicable) and the Introduction, and should be 150-200 words long. The aim should be to make your findings accessible to a wide audience that includes both scientists and non-scientists. Sample summaries can be found on our website under Submission Guidelines:

https://journals.plos.org/plosntds/s/submission-guidelines#loc-parts-of-a-submission

3) We do not publish any copyright or trademark symbols that usually accompany proprietary names, eg ©,  ®, or TM  (e.g. next to drug or reagent names). Therefore please remove all instances of trademark/copyright symbols throughout the text, including:

- ® on pages: 2, 3, 4, 5, 6, 9, 10, 11, 18, 19, and 25.

4) Please upload all main figures as separate Figure files in .tif or .eps format. For more information about how to convert and format your figure files please see our guidelines:

https://journals.plos.org/plosntds/s/figures

5) We notice that your supplementary Tables are included in the manuscript file. Please remove them and upload them with the file type 'Supporting Information'. Please ensure that each Supporting Information file has a legend listed in the manuscript after the references list.

6) In the online submission form, you indicated that We are ready to share any additional data asked by the reviewers.. All PLOS journals now require all data underlying the findings described in their manuscript to be freely available to other researchers, either

1. In a public repository

2. Within the manuscript itself

3. Uploaded as supplementary information.

This policy applies to all data except where public deposition would breach compliance with the protocol approved by your research ethics board. If your data cannot be made publicly available for ethical or legal reasons (e.g., public availability would compromise patient privacy), please explain your reasons by return email and your exemption request will be escalated to the editor for approval. Your exemption request will be handled independently and will not hold up the peer review process, but will need to be resolved should your manuscript be accepted for publication. One of the Editorial team will then be in touch if there are any issues.

7) Please amend your detailed Financial Disclosure statement. This is published with the article. It must therefore be completed in full sentences and contain the exact wording you wish to be published.

i) State the initials, alongside each funding source, of each author to receive each grant. For example: "This work was supported by the National Institutes of Health (####### to AM; ###### to CJ) and the National Science Foundation (###### to AM)."

ii) State what role the funders took in the study. If the funders had no role in your study, please state: "The funders had no role in study design, data collection and analysis, decision to publish, or preparation of the manuscript."

iii) If any authors received a salary from any of your funders, please state which authors and which funders..

If you did not receive any funding for this study, please simply state: u201cThe authors received no specific funding for this work.u201d

8) We have amended your Competing Interest statement to comply with journal style. We kindly ask that you double check the statement and let us know if anything is incorrect.

**Reviewers' Comments:**

Reviewer's Responses to Questions

**Key Review Criteria Required for Acceptance?**

As you describe the new analyses required for acceptance, please consider the following:

**Methods**

-Are the objectives of the study clearly articulated with a clear testable hypothesis stated?

-Is the study design appropriate to address the stated objectives?

-Is the population clearly described and appropriate for the hypothesis being tested?

-Is the sample size sufficient to ensure adequate power to address the hypothesis being tested?

-Were correct statistical analysis used to support conclusions?

-Are there concerns about ethical or regulatory requirements being met?

Reviewer #1: The objectives are articulated (though 'accuracy' is not listed initially, and pops up later). The design and population are appropriate. The sample size seems reasonable. Ethics approval was obtained.

• Line 86 – could there please be MUCH more information provided on what the standard stool workup was (what agar plates, what incubation, what identification, specifics of the diarrhoeagenic E coli molecular testing, specifics of the viral PCR workup, and more details of the microscopy techniques)?

• What was the interval between sample collection and storage at -80C? How long were samples stored before testing?

• Line 118 – I do not understand what this means. If a true positive is defined as both assays positive, then both conventional and 2SMART testing would both have 100% sensitivity for all targets, since both would have detected all true positives. This is contradicted by Line 135. Line 138 says that PPV was the probability that patients with a positive test ‘truly had a pathogen’ – what does ‘truly had a pathogen’ mean?

• I have real concerns about the validity of the conventional reference standard assays. It only detected 1/37 ETEC positives, 0/36 EPEC positives, 1/12 Salmonella positives, 3/34 Campylobacter positives, 7/29 Norovirus positives, and 11/72 adenovirus positives, for example. For the bacterial pathogens, this is lower than I would have expected (even given the expected superior sensitivity of molecular testing) and – for the viral pathogens – why was there such a difference if molecular is currently being used for conventional testing?

• What is the CUI, and how is it helpful to gauge performance of the assay of interest?

• How does the Bayesian latent class analysis (and the Youden index) add to our understanding of the assay of interest? This probably needs to be explained in details for readers like me who are not familiar with this analysis.

• Could you please describe the setting in more detail? Number of yearly clinic visits, number of yearly hospitalizations

• What does ‘pediatric’ mean? Age 1 day to 18 years?

• How was it decided whether to take a rectal swab or bulk stool?

• What type of rectal swabs were used? What transport medium?

• How was diagnostic accuracy calculated? Is this really helpful given the expected low sensitivity of conventional testing?

Reviewer #2: -Are the objectives of the study clearly articulated with a clear testable hypothesis stated?

The objective was clear. A testable hypothesis was not provided - yet the authors do compare the conventional assay to the multiplex qPCR

-Is the study design appropriate to address the stated objectives?

The study design is not described including the study setting. One only learns the design as they read the different sections of the manuscript and from the objective.

-Is the population clearly described and appropriate for the hypothesis being tested?

Details of the study participants are quite scanty for any meaningful reconstruction of the of the study. The study location(s) is not provided including which hospital(s) the participants were drawn from and the type of facility. Participants usage of antibiotics or other treatments prior to sample collection is not provided.

-Is the sample size sufficient to ensure adequate power to address the hypothesis being tested?

Sample size details are not provided - to get a clear understanding of how many would have been required to test for the pathogens. On line 46-48 - the authors point “Sensitivity estimates varied, with lower sensitivity observed for EPEC, Cryptosporidium, G. lamblia, and Rotavirus, partly due to the small sample numbers and wide confidence intervals.” Additionally, for the 153 individual patients- how many contributed stool and how many contributed swabs?

-Were correct statistical analysis used to support conclusions?

I have limitations in statistics - but assume that Bayesian latent class model (LCM) as applied to estimate diagnostic accuracy in the study to generates Posterior medians with 95% credible intervals. On line 150-152 authors indicate “This method considers both the tests as imperfect. We adopted a two-test one-population model assuming conditional independence. For all the parameters, we assumed a non-informative prior distribution (beta distribution (0.5,0.5))”. The authors need to be clear about potential independence in detection of nucleic acids by both approaches/assays, dependence on pathogen load, effect of recent antibiotic use on culture positivity etc which may bias estimates. The limitation of applications of such LCM approaches when applied to pathogen detection work needs careful considerations related to inherent aspects of samples sizes, pathogen prevalences, model identification, priors and assumptions.

Other concerns

1. Does the amount of starting material differ for nucleic acid extractions considering both swabs and whole stool were used? Would this influence the down-stream assays to be conducted? From a practical perspective - a swab from a diarrhoea stool sample with unusual character - line 84-85 (liquid, watery, mucous or bloody purulent) may influence how much material is collected. Since this was a “head-to-head” comparison (i.e. GastroFinder® 2SMART kit compared with conventional assay techniques - lines 76-79) was one swab split between the different targets and assays? Could this influence study outcomes because of bias considering the metrics for success - performance, sensitivity, specificity, positive predictive value (PPV), negative predictive value (NPV), and likelihood ratio (LR)?

2. Two references are provided for the routine tests for enteric bacteria by culture, PCR assay for diarrheagenic Escherichia coli, reverse-transcription PCR for enteric viruses, and microscopy for the identification of parasites (line 86-88). The study by Nair et al 2010 https://pmc.ncbi.nlm.nih.gov/articles/PMC2901208/, indicates “Faecal specimens were collected in McCartney bottles using sterile catheters or as rectal swabs in Cary Blair medium and were examined within 2 hrs for 24 enteric pathogens comprising bacterial, viral and parasitic pathogens using a combination of conventional, immunological and molecular methods” and don’t highlight use of fecal swabs. The other by Mitra et al., 2020 https://pubmed.ncbi.nlm.nih.gov/32585545/ (targeted 4 common enteric viruses (rotavirus, norovirus, adenovirus, astrovirus) but due to paywall - I could not access the methods. Conventional methods for certain pathogens such as Salmonella spp and Dientamoeba falgillis are not detailed in the study by Mitra et al are not provided. It’s also not clear why there was no effort to target the specific pathogens present in the GastroFinder® 2SMART assay - on line 44 - 46 - authors indicate “Certain pathogens, including Yersinia enterocolitica, Clostridioides difficile, STEC, and Dientamoeba fragilis were not tested in the conventional panel and therefore not considered discordant”. There is no clear explanation in the methods as to why this is the case yet the authors, concurrently, provide quite some details of the PCR from line 103 to 117 on the GastroFinder® 2SMART assay is combined with a normal PCR.

3. Does the nucleic acids extraction kits matter? The QIAamp DNA Mini Kit (Qiagen) was used for extraction of total nucleic acids and eluted in a volume of 50 μl of which 5 μl was used for PCR. There are minimal details on the procedure for extractions including whether bead beating was a necessary step considering the nature and biology of bacterial pathogens etc. The manufacturer indicates that the “validated extraction methods NucliSENS® easyMAG® (bioMérieux)” - which may simply imply that the manufacturer has not tested the Qiagen kit - so, providing extra details of the kits protocol is likely essential - and whether this would vary by swabs compared to whole stool. Sometimes, knowing the amount of microbial load maybe important - for example 16S for bacterial pathogens. Would the Kit be efficient at targeting RNA based viruses?

**********

**Results**

-Does the analysis presented match the analysis plan?

-Are the results clearly and completely presented?

-Are the figures (Tables, Images) of sufficient quality for clarity?

Reviewer #1: • Could the numbers of pathogens detected by conventional testing and the study assay also be presented (rather than simply ‘sole infection’ and ‘mixed infection’)?

• Line 121-124 – this does not appear to be methods nor results; rather, it appears to be a conclusion of the authors, and so would be better placed in the Discussion

• Line 162 – is this a description of how children are treated generally in the study setting or does it refer to the study population? If it refers to the study population, and is important, could it be made more specific?

• It seems curious that only 5% of the study population had fever. This probably deserves more comment – what was the chief complaint of the study population (ie. the reason they came to clinic or were hospitalized)? For example – were some of these children who were admitted with asthma exacerbation and just happened to have diarrhoea as well?

• Line 176-178 seems to be methods

Reviewer #2: -Does the analysis presented match the analysis plan?

Yes - partly.

-Are the results clearly and completely presented?

On line 88, authors indicate that Demographic and clinical information was also collected. However, only age and gender as well as clinical signs and symptoms (type of diarrhoea, and whether the patients had vomiting, fever, dehydration, or abdominal pain) were collected. This limited detail makes its slightly difficult to understand the study population better including demography, treatment, diagnosis, duration of illness or diarrhoea, outpatients versus inpatients, vaccination etc.

Performance, sensitivity, specificity, positive predictive value (PPV), negative predictive value (NPV), and likelihood ratio (LR) is indicated as the key focus of the study. However - only a paragraph attempts to discuss this - but in quite a scanty manner - lines 185 - 192. Also, the true data points are not presented on the text. for example - line 185 "Sensitivity for EPEC, Cryptosporidium, G. lamblia, and Rotavirus appeared low in this cohort, with wide confidence intervals". How can appearing low be judged? also line 188 - 189 "The specificity was good in detecting the majority of pathogens with fairly precise confidence intervals". Good?

-Are the figures (Tables, Images) of sufficient quality for clarity?

It’s not clear to me why the melting peaks/curves are presented here (figure 2). I may be wrong, but I assumed that this would already have been validated by the manufacturer and therefore, only needed to be checked for validity including assessing positive and negative controls as part of standard operations expected of these experiments. This would be the same assumption I would easily make for the PCR amplification details highlighted in the methods. The expected melting curves and Tm ranges should already have been established by the manufacturer during assay development and validation - internal control, amplification controls and positive controls. Manufacturer establishes primer/probe design, analytical sensitivity/specificity, expected Tm windows, fluorescence channels, controls, compatible instruments and interpretation rules. The expectation here was checking that the assay performs as expected with the selected extraction method, thermocycler, stool samples, operators and laboratory conditions. Its likely that manufacturer provides software plus internal/positive/negative controls, but laboratories do review melting curves/Tm values for unusual, weak, multiple or borderline results.

Figures: Both Figure 1 and 2 are not legible even when one zooms in. The coordinates and labels on both axis are not legible

other comments

1. The median age of the study children can be reported in months considering the summary distributions - Age in years: Median (IQR) 1.0 (0.09, 1.11).

2. The rates reported for conventional assays in line 164 - may need checking - detection rates were significantly higher with qPCR than in conventional testing (81% vs 43.8% - also in the abstract). I assumed its 41.8% i.e. 64/153 as indicated on table 2 - any pathogen positive sample by conventional method.

3. Distribution of single and multiple infections needs to be reported for both conventional and PCR based methods

4. Suggest that Table 4 be organised by types of pathogens similar to Table 3 for easy comparison.

5. Several wording and tense phrases need checking e.g. line 176 - have been detected very frequently, line 181 - has been observed. Check elsewhere.

**********

**Conclusions**

-Are the conclusions supported by the data presented?

-Are the limitations of analysis clearly described?

-Do the authors discuss how these data can be helpful to advance our understanding of the topic under study?

-Is public health relevance addressed?

Reviewer #1: I think the Discussion section could do with reworking to better place the results in the context of other studies and to better expand on the limitations of the study.

• The first three paragraphs of the discussion appears to be background information. I would recommend using the beginning of the discussion to discuss the most important results of the study.

• Line 229 – underdiagnosis of rotavirus would be a fatal flaw for an assay designed for paediatrics, given the morbidity and mortality caused by this pathogen in many parts of the world. I appreciate the authors’ having thought about WHY this might have occurred – but the impact of this deficiency needs to also be discussed in detail.

• Line 239 – this paragraph is also mostly background

• Line 246 – I think the intent of this paragraph is to compare the relative prevalence of certain pathogens in the study population with that in different areas. If so, it should be rewritten to more clearly compare and contrast so that the intended conclusions can be drawn…

Reviewer #2: Overall, the conclusions don't follow from the results and are broadly general and need careful rewording.

The limitations described are few and could easily be expanded.

The first paragraph needs a summary of the results and not background details which are covered in the introduction. Line 200 - 226 appear mostly background details. The discussion also lacks coherence and would have benefited if an initial structure was developed and followed to avoid randomness.

The focus of the study results has not been discussed - the study evaluated GastroFinder® 2SMART kit to check its performance, sensitivity, specificity, positive predictive value (PPV), negative predictive value (NPV), and likelihood ratio (LR) - line 75 - 77. Diagnostic performance measures including sensitivity, specificity, predictive values, accuracy, and probabilistic clinical utility index (CUI) were estimated with 95% confidence intervals (CI) - line 129 - 131. However, none of these is available in the discussion section. The results from the study are actually rarely discussed or interpreted to get meaningful insight what this truly means. There is a bit of detail on Rotavirus but not the other pathogens or pathogen groups.

There is a drive towards pushing GastroFinder® 2SMART assay as a molecular point-of-care testing but this possibly needs some thoughts on cost implications, feasibility, diagnostic utility etc considering the results described here - sensitivity for some of the well-known diarrheal pathogens was low - while details of certain pathogens in the population are not provided e.g. Yersinia, C. diff., STEC, etc. Could the authors highlight “the diagnostic usefulness” of the assay especially for a LMIC settings and implications for mixed infections. For this assay, what would over/underdiagnosis look like in field scenarios considering the different stages of disease and patient journeys seeking healthcare?

The concordance or discrepance of the frequentists versus the Bayesian latent class analysis on performance metrics needs to be described or compared.

**********

**Editorial and Data Presentation Modifications?**

Use this section for editorial suggestions as well as relatively minor modifications of existing data that would enhance clarity. If the only modifications needed are minor and/or editorial, you may wish to recommend “Minor Revision” or “Accept”.

Reviewer #1: (No Response)

Reviewer #2: (No Response)

**********

**Summary and General Comments**

Use this section to provide overall comments, discuss strengths/weaknesses of the study, novelty, significance, general execution and scholarship. You may also include additional comments for the author, including concerns about dual publication, research ethics, or publication ethics. If requesting major revision, please articulate the new experiments that are needed.

Reviewer #1: The authors should be commended for evaluating another multiplex assay for enteric pathogen detection, given the morbidity and mortality attributable to acute diarrhoeal disease in South Asia. However, I think major revisions to this article would be required to consider it for publication in this journal. I am unsure of exactly how much better the assay is than conventional testing given the lack of detail provided on what was ‘conventional’ in this study population and the lack of description of the study population; I am also unfamiliar with some of the metrics used to measure assay performance. Data availability seems very vague.

Reviewer #2: The study by Chowdhury and colleagues is an important one in attempting to enumerate the etiology of diarrhoea infections in young children which are linked to mortality and morbidity in many LMIC settings. They focussed on a customised commercially produced PCR based assay targeting 17 enteric pathogens (bacteria, virus, and parasites) which are players in diarrhoea aetiology. The overall outlook of the paper is that it needs quite some strengthening across the methods, results and discussion to help with interpretation.

The comments are highlighted in the specific sections above.

**********

PLOS authors have the option to publish the peer review history of their article (what does this mean?). If published, this will include your full peer review and any attached files.

If you choose “no”, your identity will remain anonymous but your review may still be made public.

**Do you want your identity to be public for this peer review?**  For information about this choice, including consent withdrawal, please see our Privacy Policy.

Reviewer #1: No

Reviewer #2: No

[NOTE: If reviewer comments were submitted as an attachment file, they will be attached to this email and accessible via the submission site. Please log into your account, locate the manuscript record, and check for the action link "View Attachments". If this link does not appear, there are no attachment files.]

**Figure resubmission:**

While revising your submission, we strongly recommend that you use PLOS’s NAAS tool (https://ngplosjournals.pagemajik.ai/artanalysis) to test your figure files. NAAS can convert your figure files to the TIFF file type and meet basic requirements (such as print size, resolution), or provide you with a report on issues that do not meet our requirements and that NAAS cannot fix.

After uploading your figures to PLOS’s NAAS tool - https://ngplosjournals.pagemajik.ai/artanalysis, NAAS will process the files provided and display the results in the "Uploaded Files" section of the page as the processing is complete. If the uploaded figures meet our requirements (or NAAS is able to fix the files to meet our requirements), the figure will be marked as "fixed" above. If NAAS is unable to fix the files, a red "failed" label will appear above. When NAAS has confirmed that the figure files meet our requirements, please download the file via the download option, and include these NAAS processed figure files when submitting your revised manuscript.

**Reproducibility:**

To enhance the reproducibility of your results, we recommend that authors of applicable studies deposit laboratory protocols in protocols.io, where a protocol can be assigned its own identifier (DOI) such that it can be cited independently in the future. Additionally, PLOS ONE offers an option to publish peer-reviewed clinical study protocols. Read more information on sharing protocols at https://plos.org/protocols?utm_medium=editorial-email&utm_source=authorletters&utm_campaign=protocols

# Author comment: Author response to Decision Letter 1

**DOI:** 10.1371/journal.pntd.0014823.r002

14 Aug 2026

Attachment Submitted filename: Response to the Comments.docx

# Peer review: Decision Letter 1

**DOI:** 10.1371/journal.pntd.0014823.r003

**Authors:** Mathieu Picardeau, Kirkby Tickell

22 Sep 2026

PNTD-D-26-00570R1

Enhancing Pathogen Detection in Pediatric Diarrheal Stools: The Case of Molecular Multiplexing with a Real-time PCR Assay

PLOS Neglected Tropical Diseases

Dear Dr. Ramamurthy,

Thank you for submitting your manuscript to PLOS Neglected Tropical Diseases. After careful consideration, we feel that it has merit but does not fully meet PLOS Neglected Tropical Diseases's publication criteria as it currently stands. Therefore, we invite you to submit a revised version of the manuscript that addresses the points raised during the review process.

Please submit your revised manuscript within by Nov 21 2026 11:59PM. If you will need more time than this to complete your revisions, please reply to this message or contact the journal office at plosntds@plos.org. When you're ready to submit your revision, log on to https://www.editorialmanager.com/pntd/ and select the 'Submissions Needing Revision' folder to locate your manuscript file.

Please include the following items when submitting your revised manuscript:

* A letter that responds to each point raised by the editor and reviewer(s). You should upload this letter as a separate file labeled 'Response to Reviewers'. This file does not need to include responses to any formatting updates and technical items listed in the 'Journal Requirements' section below.

* A marked-up copy of your manuscript that highlights changes made to the original version. You should upload this as a separate file labeled 'Revised Manuscript with Track Changes'.

* An unmarked version of your revised paper without tracked changes. You should upload this as a separate file labeled 'Manuscript'.

If you would like to make changes to your financial disclosure, competing interests statement, or data availability statement, please make these updates within the submission form at the time of resubmission. Guidelines for resubmitting your figure files are available below the reviewer comments at the end of this letter.

As the corresponding author, your ORCID iD is verified in the submission system and will appear in the published article. PLOS supports the use of ORCID, and we encourage all coauthors to register for an ORCID iD and use it as well. Please encourage your coauthors to verify their ORCID iD within the submission system before final acceptance, as unverified ORCID iDs will not appear in the published article. Only the individual author can complete the verification step; PLOS staff cannot verify ORCID iDs on behalf of authors.

We look forward to receiving your revised manuscript.

Kind regards,

Kirkby D Tickell, MBBS BSc

Academic Editor

PLOS Neglected Tropical Diseases

Mathieu Picardeau

Section Editor

PLOS Neglected Tropical Diseases

Shaden Kamhawi

co-Editor-in-Chief

PLOS Neglected Tropical Diseases

orcid.org/0000-0003-4304-636XX

Paul Brindley

co-Editor-in-Chief

PLOS Neglected Tropical Diseases

orcid.org/0000-0003-1765-0002

**Journal Requirements:**

If the reviewer comments include a recommendation to cite specific previously published works, please review and evaluate these publications to determine whether they are relevant and should be cited. There is no requirement to cite these works unless the editor has indicated otherwise.

1) Please ensure that the CRediT author contributions listed for every co-author are completed accurately and in full.

At this stage, the following Authors/Authors require contributions: Goutam Chowdhury, Melissa Glenda Lewis, Alok Deb, Mamta Chawla-Sarkar, Sandipan Ganguly, Paulami Dutta, Santasabuj Das, Asish K. Mukhopadhyay, and Thandavarayan Ramamurthy. Please ensure that the full contributions of each author are acknowledged in the "Add/Edit/Remove Authors" section of our submission form.

The list of CRediT author contributions may be found here: https://journals.plos.org/plosntds/s/authorship#loc-author-contributions

2) Please amend your detailed Financial Disclosure statement. This is published with the article. It must therefore be completed in full sentences and contain the exact wording you wish to be published.

i) State the initials, alongside each funding source, of each author to receive each grant. For example: "This work was supported by the National Institutes of Health (####### to AM; ###### to CJ) and the National Science Foundation (###### to AM).".

If you did not receive any funding for this study, please simply state: u201cThe authors received no specific funding for this work.u201d

**Reviewers' Comments:**

Reviewer's Responses to Questions

**Key Review Criteria Required for Acceptance?**

As you describe the new analyses required for acceptance, please consider the following:

**Methods**

-Are the objectives of the study clearly articulated with a clear testable hypothesis stated?

-Is the study design appropriate to address the stated objectives?

-Is the population clearly described and appropriate for the hypothesis being tested?

-Is the sample size sufficient to ensure adequate power to address the hypothesis being tested?

-Were correct statistical analysis used to support conclusions?

-Are there concerns about ethical or regulatory requirements being met?

Reviewer #1: • Exclusion criteria – you do not need to specify criteria that are the inverse of inclusion criteria…just add the exclusions that are applicable AFTER following inclusion criteria

• Would specify how many stools were collected and then how many were excluded (because of sample quality, previous antibiotic use, etc) to get to the final number of 153

• I am still not sure that the CUI values add anything beyond what one would understand looking at sensitivity, specificity, PPV, and NPV. I am freely willing to believe there MIGHT be a benefit – but, if there is, it has not been explained. Is there a reference for the definition of CUI for diagnostic tests? Is there a reference for the grading (excellent-good-fair-poor-very poor) of the CUI? In contrast, I do not think sensitivity, specificity, PPV, and NPV need to be defined (lines 224-229), as these are very common terms.

Reviewer #2: -Are the objectives of the study clearly articulated with a clear testable hypothesis stated? The objectives have now been outlined and the authors indicate that No formal a priori sample size calculation was performed because the study was designed as an initial evaluation of the diagnostic performance of the GastroFinder 2SMART test

-Is the study design appropriate to address the stated objectives? Yes

-Is the population clearly described and appropriate for the hypothesis being tested? The authors have now provided details about the study population to help with study reconstruction including inclusion and exclusion criteria, and sampling. The population is appropriate - children with diarrheal disease.

-Is the sample size sufficient to ensure adequate power to address the hypothesis being tested? This is somewhat hard to gauge as a power calculation was not included - but authors emphasize that the objective was initial evaluation of diagnostic performance of the GastroFinder 2SMART test for which the number of children included looks sufficient.

-Were correct statistical analysis used to support conclusions? Am limited in statistics but most of the questions raised around the head-to-head comparison and the Bayesian framework have been addressed.

-Are there concerns about ethical or regulatory requirements being met? The study was conducted during 2020-2022 at the Dr. B. C. Roy Memorial Hospital for Children, Kolkata, India, which is a major government pediatric hospital providing specialised care for infants and children. They indicate that the work was approved by the Institutional Ethical Committee (IEC) of the Indian Council of Medical Research-National Institute for Research in Bacterial Infections (ICMR-NIRBI), Kolkata, India (approval no. A-1/2015-IEC). So, no concerns.

**********

**Results**

-Does the analysis presented match the analysis plan?

-Are the results clearly and completely presented?

-Are the figures (Tables, Images) of sufficient quality for clarity?

Reviewer #1: • Lines 262-264 – this information about rotavirus vaccine and uptake could be included with the rest of the description of the setting in the Methods

• There were 25 STEC detected?? I find that very surprising that there were more STEC than other pathogens that I would have thought to be much more common (including Salmonella, Shigella, rotavirus, EPEC). Why was this not included in the table? One probably should not say that they are ‘infrequently encountered’ if they are frequently encountered…

• I still am not sure how the Youden Index helps the reader understand here. The Youden Index will be optimized when sensitivity and specificity are both high…but since the ‘probable true’ sensitivity of the reference is low, specificity for multiple targets calculates to be much lower than it should be in ‘reality’. (For example, I bet the ‘true’ specificity of the campy assay is much higher than 79%.) If specificity is artificially lowered, then the Youden Index is artificially lowered too. The Youden is best for Shigella probably because the conventional testing is least bad for this organism…but isn’t the point of this article that conventional testing is terrible for a number of targets? Wouldn’t it be easier just to present McNemar P values showing that the 2SMART and conventional don’t agree for very many targets? (I think most readers realize that molecular sensitivity for stool testing for many targets is better than culture or immunoassay.)

Reviewer #2: -Does the analysis presented match the analysis plan? Largely - yes, they address the objectives in a stepwise manner.

-Are the results clearly and completely presented? Yes, the tables for the results are provided and interpreted.

-Are the figures (Tables, Images) of sufficient quality for clarity? Tables are clear and previous figures have been dropped based on the first review.

**********

**Conclusions**

-Are the conclusions supported by the data presented?

-Are the limitations of analysis clearly described?

-Do the authors discuss how these data can be helpful to advance our understanding of the topic under study?

-Is public health relevance addressed?

Reviewer #1: • Line 317: Is the main thrust of the manuscript that the 2SMART assay is much better than conventional methods or that real-time PCR is better than conventional methods? This should be clarified. Again – I am not sure that the 2SMART assay would be better than other non-multiplexed real-time viral assays…and the 2SMART has serious limitations for use in children in LMICs if it cannot detect rotavirus properly, given that this is one of the MOST important pathogens. The authors do a good job of explaining the significance of rotavirus lower down – but it probably deserves to also be included in the first sentence of the discussion.

• Line 325 – whether the testing improves antimicrobial therapy use is dependent on a number of different factors, including: timing of testing; whether antimicrobials are used at admission for diarrhoea or for other illnesses (many children hospitalized for pneumonia [for example] at the study hospital also probably have diarrhoea!); whether clinicians would be receptive to changing therapy based on test results; availability of different antibiotics; etc

• Line 333 – this reference should probably be replaced by the updated GBD paper published in Lancet ID in June 2026

• Line 352 – the STEC detection rates should probably be discussed

• Line 376 – it is not novel that molecular diagnostics have good NPV for enteric infections

• Line 381 – the sensitivity of 87% of the 2SMART for noro detection compared to conventional methods does not seem that good

• Line 384 – why would poor specificity of the adeno target in THIS cohort be due to asymptomatic carriage? The underlying assumption in this study was that all had infectious diarrhoeal disease

• Bayesian section needs to be reviewed by a statistician

• Line 438 – is the 2SMART harder to stand up in an LMIC setting than a micro lab capable of doing culture and other traditional techniques (eg TSI)?

• Line 453 – it is stated ‘enrolled patients presented with diarrhoea as their main symptom.’ How is this known? The eligibility criteria do not state anything about excluding children who had additional non-diarrhoeal diagnoses or whose main reason for admission to hospital was not diarrhoea. If that IS the case – the eligibility criteria should be revised.

Reviewer #2: -Are the conclusions supported by the data presented? I find the word “significant” - line 487 and “excellent” line 489 to be somewhat an overstatement based on the assay limitations. This language can be moderated in the final versions.

-Are the limitations of analysis clearly described? Yes - this has been well addressed.

-Do the authors discuss how these data can be helpful to advance our understanding of the topic under study? They provide some evidence that the tool under investigation can enhance pathogen detection in pediatric diarrheal stools, with an overall detection rate 2-fold higher than conventional methods. The assay shows excellent higher/better diagnostic performance for certain pathogens, supporting its use in clinical settings.

-Is public health relevance addressed? The authors state that this molecular approach has the potential to improve patient management, support antimicrobial stewardship, and enhance surveillance of diarrheal disease epidemiology. Future studies should evaluate the cost-effectiveness of this assay in LMIC settings and its impact on clinical outcomes.

**********

**Editorial and Data Presentation Modifications?**

Use this section for editorial suggestions as well as relatively minor modifications of existing data that would enhance clarity. If the only modifications needed are minor and/or editorial, you may wish to recommend “Minor Revision” or “Accept”.

Reviewer #1: • The first line of the abstract is still incorrect. Diarrhoea is not an infectious disease by definition (many noninfectious pathologies can cause diarrhoea)

• Line 92 – the URL should probably be placed in the reference list

• Line 106 – consider using ‘high-income’ instead of ‘developed’ countries

Reviewer #2: The revised version has greatly improved the manuscript - the discussion sections can have the subtitles withdrawn but leave limitations and conclusions subheadings.

**********

**Summary and General Comments**

Use this section to provide overall comments, discuss strengths/weaknesses of the study, novelty, significance, general execution and scholarship. You may also include additional comments for the author, including concerns about dual publication, research ethics, or publication ethics. If requesting major revision, please articulate the new experiments that are needed.

Reviewer #1: Overall, I still think this is a useful study, though the main thrust - molecular diagnostics are more sensitive than conventional diagnostics for the detection of diarrhoeal pathogens - is not novel. I would say that this assay itself should not be used for the evaluation of paediatric diarrhoeal disease if the rotavirus target is quite insensitive, given the importance of this pathogen. (The authors do discuss this at length, which is a significant improvement from previously.)

Reviewer #2: ROUND 1

Comments

Recommendation is major/substantial revision

The study by Chowdhury and colleagues is quite an important one in attempting to enumerate the etiology of diarrhoea infections in young children which are linked to mortality and morbidity in many LMIC settings. They focussed on a customised commercially produced PCR based assay targeting 17 enteric pathogens (bacteria, virus, and parasites) which are players in diarrhoea aetiology. The overall outlook of the paper is that it needs quite some strengthening across the methods, results and discussion to help with interpretation.

Some major comments below

Introduction

There needs to be some background on GastroFinder® 2SMART the focus of the study - Why the choice of this specific kit unlike other molecular detections out there and where else it has been used especially in LMICs.

Materials and Methods:

1. Details of the study participants are quite scanty for any meaningful reconstruction of the of the study. The study location(s) is not provided including which hospital(s) the participants were drawn from and the type of facility. Participants usage of antibiotics or other treatments prior to sample collection is not provided.

2. Sample size details are not provided - to get a clear understanding of how many would have been required to test for the pathogens. On line 46-48 - the authors point “Sensitivity estimates varied, with lower sensitivity observed for EPEC, Cryptosporidium, G. lamblia, and Rotavirus, partly due to the small sample numbers and wide confidence intervals.” Additionally, for the 153 individual patients- how many contributed stool and how many contributed swabs?

3. Does the amount of starting material differ for nucleic acid extractions considering both swabs and whole stool were used? Would this influence the down-stream assays to be conducted? From a practical perspective - a swab from a diarrhoea stool sample with unusual character - line 84-85 (liquid, watery, mucous or bloody purulent) may influence how much material is collected. Since this was a “head-to-head” comparison (i.e. GastroFinder® 2SMART kit compared with conventional assay techniques - lines 76-79) was one swab split between the different targets and assays? Could this influence study outcomes because of bias considering the metrics for success - performance, sensitivity, specificity, positive predictive value (PPV), negative predictive value (NPV), and likelihood ratio (LR)?

4. Two references are provided for the routine tests for enteric bacteria by culture, PCR assay for diarrheagenic Escherichia coli, reverse-transcription PCR for enteric viruses, and microscopy for the identification of parasites (line 86-88). The study by Nair et al 2010 https://pmc.ncbi.nlm.nih.gov/articles/PMC2901208/, indicates “Faecal specimens were collected in McCartney bottles using sterile catheters or as rectal swabs in Cary Blair medium and were examined within 2 hrs for 24 enteric pathogens comprising bacterial, viral and parasitic pathogens using a combination of conventional, immunological and molecular methods” and don’t highlight use of fecal swabs. The other by Mitra et al., 2020 https://pubmed.ncbi.nlm.nih.gov/32585545/ (targeted 4 common enteric viruses (rotavirus, norovirus, adenovirus, astrovirus) but due to paywall - I could not access the methods. Conventional methods for certain pathogens such as Salmonella spp and Dientamoeba falgillis are not detailed in the study by Mitra et al are not provided. It’s also not clear why there was no effort to target the specific pathogens present in the GastroFinder® 2SMART assay - on line 44 - 46 - authors indicate “Certain pathogens, including Yersinia enterocolitica, Clostridioides difficile, STEC, and Dientamoeba fragilis were not tested in the conventional panel and therefore not considered discordant”. There is no clear explanation in the methods as to why this is the case yet the authors, concurrently, provide quite some details of the PCR from line 103 to 117 on the GastroFinder® 2SMART assay is combined with a normal PCR.

5. Does the nucleic acids extraction kits matter? The QIAamp DNA Mini Kit (Qiagen) was used for extraction of total nucleic acids and eluted in a volume of 50 μl of which 5 μl was used for PCR. There are minimal details on the procedure for extractions including whether bead beating was a necessary step considering the nature and biology of bacterial pathogens etc. The manufacturer indicates that the “validated extraction methods NucliSENS® easyMAG® (bioMérieux)” - which may simply imply that the manufacturer has not tested the Qiagen kit - so, providing extra details of the kits protocol is likely essential - and whether this would vary by swabs compared to whole stool. Sometimes, knowing the amount of microbial load maybe important - for example 16S for bacterial pathogens. Would the Kit be efficient at targeting RNA based viruses?

6. I have limitations in statistics - but assume that Bayesian latent class model (LCM) as applied to estimate diagnostic accuracy in the study to generates Posterior medians with 95% credible intervals. On line 150-152 authors indicate “This method considers both the tests as imperfect. We adopted a two-test one-population model assuming conditional independence. For all the parameters, we assumed a non-informative prior distribution (beta distribution (0.5,0.5))”. The authors need to be clear about potential independence in detection of nucleic acids by both approaches/assays, dependence on pathogen load, effect of recent antibiotic use on culture positivity etc which may bias estimates. The limitation of applications of such LCM approaches when applied to pathogen detection work needs careful considerations related to inherent aspects of samples sizes, pathogen prevalences, model identification, priors and assumptions.

Results

1. On line 88, they indicate that Demographic and clinical information was also collected. However, only age and gender as well as clinical signs and symptoms (type of diarrhoea, and whether the patients had vomiting, fever, dehydration, or abdominal pain) were collected. This limited detail makes its slightly difficult to understand the study population better including demography, treatment, diagnosis, duration of illness or diarrhoea, outpatients versus inpatients, vaccination etc.

2. It’s not clear to me why the melting peaks/curves are presented here (figure 2). I may be wrong, but I assumed that this would already have been validated by the manufacturer and therefore, only needed to be checked for validity including assessing positive and negative controls as part of standard operations expected of these experiments. This would be the same assumption I would easily make for the PCR amplification details highlighted in the methods. The expected melting curves and Tm ranges should already have been established by the manufacturer during assay development and validation - internal control, amplification controls and positive controls. Manufacturer establishes primer/probe design, analytical sensitivity/specificity, expected Tm windows, fluorescence channels, controls, compatible instruments and interpretation rules. The expectation here was checking that the assay performs as expected with the selected extraction method, thermocycler, stool samples, operators and laboratory conditions. Its likely that manufacturer provides software plus internal/positive/negative controls, but laboratories do review melting curves/Tm values for unusual, weak, multiple or borderline results.

Discussion

1. The first paragraph needs a summary of the results and not background details which are covered in the introduction. Line 200 - 226 appear mostly background details.

2. There is a drive towards pushing GastroFinder® 2SMART assay as a molecular point-of-care testing but this possibly needs some thoughts on cost implications, feasibility, diagnostic utility etc considering the results described here - sensitivity for some of the well-known diarrheal pathogens was low - while details of certain pathogens in the population are not provided e.g. Yersinia, C. diff., STEC, etc. Could the authors highlight “the diagnostic usefulness” of the assay especially for a LMIC settings and implications for mixed infections. For this assay, what would over/underdiagnosis look like in field scenarios considering the different stages of disease and patient journeys seeking healthcare?

3. The concordance or discrepance of the frequentists versus the Bayesian latent class analysis on performance metrics needs to be described or compared.

Minor comments

Abstract: line 41: Panel breadth would not arise since only pathogens detected in both assays were compared.

Introduction

Materials and Methods

1. Some texts appear in the methods appear to provide details that should be in the discussion or be reworded. For example - line 121 - 124 “The high NPV value of these multiplex tests prompt that they can be used as screening tools, especially during outbreaks. Although the clinical efficacy of multiplex PCR tests are to be further explored, applying these tests has the potential to improve the diagnosis of infectious gastroenteritis”.

2. Provide some refs where the Bayesian latent class model (LCM) has been applied to estimate diagnostic accuracy - and

Results

1. The median age of the study children can be reported in months considering the summary distributions - Age in years: Median (IQR) 1.0 (0.09, 1.11).

2. The rates reported for conventional assays in line 164 - may need checking - detection rates were significantly higher with qPCR than in conventional testing (81% vs 43.8% - also in the abstract). I assumed its 41.8% i.e. 64/153 as indicated on table 2 - any pathogen positive sample by conventional method.

3. Distribution of single and multiple infections needs to be reported for both conventional and PCR based methods

4. Suggest that Table 4 be organised by types of pathogens similar to Table 3 for easy comparison.

5. Several wording and tense phrases need checking e.g. line 176 - have been detected very frequently, line 181 - has been observed. Check elsewhere.

Figures: Both Figure 1 and 2 are not legible even when one zooms in. The coordinates and labels on both axis are not legible

The authors for the manuscript PNTD-D-26-00570R1 addressed the comments that I had raised and happy with the new draft. I believe it’s now quite improved and can be published. They need to remove the subtitles in the discussion.

**********

PLOS authors have the option to publish the peer review history of their article (what does this mean?). If published, this will include your full peer review and any attached files.

If you choose “no”, your identity will remain anonymous but your review may still be made public.

**Do you want your identity to be public for this peer review?**  For information about this choice, including consent withdrawal, please see our Privacy Policy.

Reviewer #1: No

Reviewer #2: **Yes:**  James M Njunge

[NOTE: If reviewer comments were submitted as an attachment file, they will be attached to this email and accessible via the submission site. Please log into your account, locate the manuscript record, and check for the action link "View Attachments". If this link does not appear, there are no attachment files.]

**Figure resubmission:**

While revising your submission, we strongly recommend that you use PLOS’s NAAS tool (https://ngplosjournals.pagemajik.ai/artanalysis) to test your figure files. NAAS can convert your figure files to the TIFF file type and meet basic requirements (such as print size, resolution), or provide you with a report on issues that do not meet our requirements and that NAAS cannot fix.

After uploading your figures to PLOS’s NAAS tool - https://ngplosjournals.pagemajik.ai/artanalysis, NAAS will process the files provided and display the results in the "Uploaded Files" section of the page as the processing is complete. If the uploaded figures meet our requirements (or NAAS is able to fix the files to meet our requirements), the figure will be marked as "fixed" above. If NAAS is unable to fix the files, a red "failed" label will appear above. When NAAS has confirmed that the figure files meet our requirements, please download the file via the download option, and include these NAAS processed figure files when submitting your revised manuscript.

**Reproducibility:**

To enhance the reproducibility of your results, we recommend that authors of applicable studies deposit laboratory protocols in protocols.io, where a protocol can be assigned its own identifier (DOI) such that it can be cited independently in the future. Additionally, PLOS ONE offers an option to publish peer-reviewed clinical study protocols. Read more information on sharing protocols at https://plos.org/protocols?utm_medium=editorial-email&utm_source=authorletters&utm_campaign=protocols

# Author comment: Author response to Decision Letter 2

**DOI:** 10.1371/journal.pntd.0014823.r004

5 Oct 2026

Attachment Submitted filename: Response to Reviewers comments-2.docx

# Editor report: Decision Letter 2

**DOI:** 10.1371/journal.pntd.0014823.r005

**Authors:** Mathieu Picardeau, Kirkby Tickell

14 Oct 2026

Dear Dr. Ramamurthy,

We are pleased to inform you that your manuscript 'Enhancing Pathogen Detection in Pediatric Diarrheal Stools: The Case of Molecular Multiplexing with a Real-time PCR Assay' has been provisionally accepted for publication in PLOS Neglected Tropical Diseases.

Before your manuscript can be formally accepted you will need to complete some formatting changes, which you will receive in a follow up email. A member of our team will be in touch with a set of requests.

Please note that your manuscript will not be scheduled for publication until you have made the required changes, so a swift response is appreciated.

IMPORTANT: The editorial review process is now complete. PLOS will only permit corrections to spelling, formatting or significant scientific errors from this point onwards. Requests for major changes, or any which affect the scientific understanding of your work, will cause delays to the publication date of your manuscript.

Should you, your institution's press office or the journal office choose to press release your paper, you will automatically be opted out of early publication. We ask that you notify us now if you or your institution is planning to press release the article. All press must be co-ordinated with PLOS.

Thank you again for supporting Open Access publishing; we are looking forward to publishing your work in PLOS Neglected Tropical Diseases.

Best regards,

Kirkby D Tickell, MBBS BSc

Academic Editor

PLOS Neglected Tropical Diseases

Mathieu Picardeau

Section Editor

PLOS Neglected Tropical Diseases

Shaden Kamhawi

co-Editor-in-Chief

PLOS Neglected Tropical Diseases

orcid.org/0000-0003-4304-636XX

Paul Brindley

co-Editor-in-Chief

PLOS Neglected Tropical Diseases

orcid.org/0000-0003-1765-0002

***********************************************************

# Editor report: Acceptance letter

**DOI:** 10.1371/journal.pntd.0014823.r006

**Authors:** Mathieu Picardeau, Kirkby Tickell

Dear Dr. Ramamurthy,

We are delighted to inform you that your manuscript, "Enhancing Pathogen Detection in Pediatric Diarrheal Stools: The Case of Molecular Multiplexing with a Real-time PCR Assay," has been formally accepted for publication in PLOS Neglected Tropical Diseases.

We have now passed your article onto the PLOS Production Department who will complete the rest of the publication process. All authors will receive a confirmation email upon publication.

The corresponding author will soon be receiving a typeset proof for review, to ensure errors have not been introduced during production. Please review the PDF proof of your manuscript carefully, as this is the last chance to correct any scientific or type-setting errors. Please note that major changes, or those which affect the scientific understanding of the work, will likely cause delays to the publication date of your manuscript. Note: Proofs for Front Matter articles (Editorial, Viewpoint, Symposium, Review, etc...) are generated on a different schedule and may not be made available as quickly.

Soon after your final files are uploaded, the early version of your manuscript will be published online unless you opted out of this process. The date of the early version will be your article's publication date. The final article will be published to the same URL, and all versions of the paper will be accessible to readers.

For Research Articles, you will receive an invoice from PLOS for your publication fee after your manuscript has reached the completed accept phase. If you receive an email requesting payment before acceptance or for any other service, this may be a phishing scheme. Learn how to identify phishing emails and protect your accounts at https://explore.plos.org/phishing.

Thank you again for supporting open-access publishing; we are looking forward to publishing your work in PLOS Neglected Tropical Diseases.

Best regards,

Shaden Kamhawi

co-Editor-in-Chief

PLOS Neglected Tropical Diseases

Paul Brindley

co-Editor-in-Chief

PLOS Neglected Tropical Diseases
